# Millennial soil retention of terrestrial organic matter deposited in the Bengal Fan

**DOI:** 10.1038/s41598-018-30091-8

**Published:** 2018-08-10

**Authors:** Katherine L. French, Christopher J. Hein, Negar Haghipour, Lukas Wacker, Hermann R. Kudrass, Timothy I. Eglinton, Valier Galy

**Affiliations:** 1 Department of Marine Chemistry and Geochemistry, Woods Hole Oceanographic Institution, 360 Woods Hole Road, Woods Hole, MA 02543 USA; 2 Department of Physical Sciences, Virginia Institute of Marine Science, College of William and Mary, P.O. Box 1346, Gloucester Point, VA 23062 USA; 3 Geological Institute, NO G59, Department of Earth Sciences, Sonneggstrasse 5, ETH, 8092 Zurich, Switzerland; 4 Laboratory for Ion Beam Physics, Department of Physics, Otto-Stern-Weg 5, ETH, 8093 Zurich, Switzerland; 5 MARUM, University of Bremen, Leobener Strasse 8, 28359 Bremen, Germany; 6 Present Address: Central Energy Resources Science Center, U.S. Geological Survey, Denver, CO 80225 USA

## Abstract

The abundance of organic carbon (OC) in vegetation and soils (~2,600 PgC) compared to carbon in the atmosphere (~830 PgC) highlights the importance of terrestrial OC in global carbon budgets. The residence time of OC in continental reservoirs, which sets the rates of carbon exchange between land and atmosphere, represents a key uncertainty in global carbon cycle dynamics. Retention of terrestrial OC can also distort bulk OC- and biomarker-based paleorecords, yet continental storage timescales remain poorly quantified. Using “bomb” radiocarbon (^14^C) from thermonuclear weapons testing as a tracer, we model leaf-wax fatty acid and bulk OC ^14^C signatures in a river-proximal marine sediment core from the Bay of Bengal in order to constrain OC storage timescales within the Ganges-Brahmaputra (G-B) watershed. Our model shows that 79–83% of the leaf-waxes in this core were stored in continental reservoirs for an average of 1,000–1,200 calendar years, while the remainder was stored for an average of 15 years. This age structure distorts high-resolution organic paleorecords across geologically rapid events, highlighting that compound-specific proxy approaches must consider storage timescales. Furthermore, these results show that future environmental change could destabilize large stores of old - yet reactive - OC currently stored in tropical basins.

## Introduction

The age of terrestrial organic components in marine sediments reflects their residence times in continental reservoirs. Rivers supply OC with a spectrum of ages to the ocean, and distinct transport pathways impart unique ages to different higher plant-derived compound classes^1–4^. This study aims to quantify the storage history of a classic terrestrial biomarker compound class, leaf-wax fatty acids^5^, and, by extension, bulk terrestrial OC in the globally significant G-B river system. The G-B rivers drain the Himalayas and deliver 1–2 billion tons of sediment annually to the Bay of Bengal, building the world’s largest delta and submarine fan: the Bengal Fan^6,7^. The G-B river system is one of the largest outlets of terrestrial OC to the ocean, with 10–20% of annual terrestrial OC burial in global marine sediments occurring in the Bengal Basin^8,9^. The G-B rivers’ extensive catchment area (1.73 × 10^6^ km^2^), steep topography, and seasonal monsoon lead to high sedimentation rates and efficient OC preservation in the Bengal Fan, where sediment effectively derives from a single source (the G-B rivers) and marine OC contributions are negligible^8,10–12^. Consequently, the Bengal Fan is an ideal location to generate high-resolution sedimentary records to study timescales of continental OC storage. While previous work has identified several components of the bulk OC possessing different sources and average ages within the G-B watershed^3^ (e.g., petrogenic C, bulk biospheric C, etc.), the age spectrum of the terrestrial OC, and more specifically leaf-wax fatty acids, delivered to the Bengal Fan has not been fully resolved and quantified.

An 18 m-long piston sediment core (SO188–336KL) was collected in 2006 at the head of the canyon that bisects the Bengal shelf^13^ (Supplementary Fig. S1). This core spans 1945 to 2006 CE according to age models derived from ^137^Cs records and correlation of storm-derived deposits with known cyclonic events (Supplementary Figs. S2 and S3). Consequently, this core captures the “bomb spike” derived from thermonuclear weapons testing, which was most active above ground in the 1950s and early 1960s. The core exhibits an exceptionally high average sedimentation rate of 30.5 cm/yr. Sedimentary units are composed of 2- to 15-cm thick, laminated sand and silt layers grading into finely laminated silty clay that are deposited during cyclone impacts of the inner shelf. Bulk geochemical data imply that the G-B rivers supply the fine-grained sediments deposited at the head of the canyon, and the sediment source was constant over the sampling interval (Supplemental Table S1)^14,15^. Large storm events could remobilize shelf-deposited sediment associated with older organic matter. Accordingly, the fine-grained (median grain size: 11–25 μm) upper units of the graded beds were selected for detailed geochemical analyses in order to avoid more heavily reworked sediment associated with coarser tempestite units.

This study focuses on saturated, even-numbered, straight-chained fatty acids, where the *n*-C_X:0_ fatty acid will be referred to as *n*-C_X_ (x corresponds to the carbon chain length). The fatty acids from each sediment horizon exhibit a strong even-over-odd carbon number predominance (carbon preference index [CPI]: 3.7–4.2) with a biomodal distribution. The long-chain fatty acid (*n*-C_24−32_) concentrations dominate over the short-chain fatty acids, with the exception of *n*-C_16_ (Supplementary Fig. S6). The *n*-C_24−32_ δ^13^C values (−25.9 to −29.1‰) are comparable to those of *n*-C_14_, *n*-C_16_, and *n*-C_22_ fatty acids (−25.8 to −29.9‰) (Supplementary Fig. S7). In contrast, *n*-C_18_ and *n*-C_20_ fatty acids are shifted towards more ^13^C-enriched values (−23.4 to −27.8‰), consistent with increased marine plankton and/or freshwater algal inputs. The long-chain fatty acid distributions and δ^13^C values are characteristic of leaf-waxes from a mixed C3/C4 plant source^5,16^, which are consistent with previous findings^17^. The Bengal Fan long-chain fatty acid δ^13^C values are more comparable to fatty acids in the lower G-B river sediments than the Himalayan river sediments^17,18^, suggesting that floodplain fatty acids are preferentially exported over fatty acids sourced from the upland regions of the catchment area.

**Figure 1:**
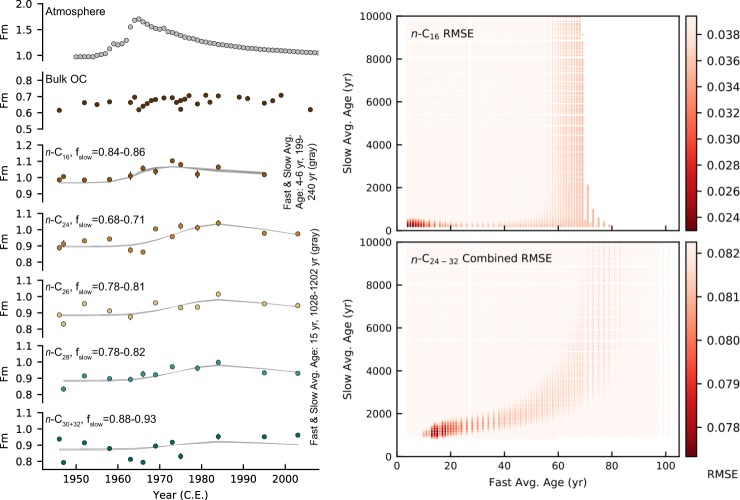
Measured radiocarbon time series and fatty acid age structure modeling results. The measured ^14^C composition of bulk OC and fatty acids reported in Fm with 1σ error bars are plotted compared to the atmospheric radiocarbon composition in the northern hemisphere zone 3^26^ (left). The heat maps (right) show how well each age structure fits the measured fatty acid Fm data according to RMSE. The color bars are scaled to bracket the RMSE values of the top 10% best-fitting age structures, where darker red represents better fits. Fast-cycling average ages exceeding 70–80 years are excluded for *n*-C_16_ because the optimal calculated f_Slow_ is outside of the allowed range of 0 to 1. Note, that the modeling results shown for C_24+_ fatty acids are based on keeping the fast- and slow-cycling endmembers constant for all of the long-chain fatty acids. Synthetic Fm time series for the eight best-fitting age distributions are plotted in gray against the measured fatty acid Fm (left). The age distribution characteristics of the *n*-C_16_ and long-chain fatty acid synthetic Fm time series are described in the first and last rows of Table 1, respectively. See Figs. S9 and S10 for RMSE heat maps over the complete slow-cycling average age range (0–50,000 years) and additional synthetic Fm time series plots.

The radiocarbon time series show that the fatty acids incorporate bomb ^14^C (Fig. 1), where the data are expressed as the fraction of modern carbon, Fm (where 0 is radiocarbon-dead). The *n*-C_16_ fatty acid Fm peaks earlier than *n*-C_24_, *n*-C_26_, and *n*-C_28_. In contrast, *n*-C_30+32_ fatty acid does not display a clear bomb spike. The fatty acid Fm excursions are significantly smaller in magnitude and delayed in time relative to the atmospheric bomb spike. Fatty acids generally become more ^14^C-depleted with increasing chain length, which is consistent with previous studies^2,19–21^. Bulk OC is systematically more ^14^C-depleted than the corresponding fatty acids.

Bulk OC radiocarbon compositions average the ages of its many constituents, including the distinct ages carried by petrogenic carbon from bedrock erosion or by different classes of plant biomarkers, such as *n*-alkanes, fatty acids, and lignin derivatives^1–3^. In contrast, a narrow age distribution centered at the measured radiocarbon age is assumed in the traditional interpretation of compound-specific radiocarbon data owing to greater source specificity. However, recent research suggests that sedimentary compound-specific radiocarbon ages are more complex than previously thought^4,22,23^.

The muted bomb spikes recorded in the fatty acid radiocarbon records demonstrate that, despite source-specificity, each fatty acid homologue possesses a complex age distribution reflecting a diversity of storage and transport timescales^4^. The pre-bomb long-chain fatty acid radiocarbon ages range from 360 to 1,860 radiocarbon years. The atmospheric ^14^C signature is incorporated at the time of biosynthesis, starting the radiocarbon clock, which then decays during storage and remobilization on the path to deposition in marine sediments. If the fatty acids were hundreds to thousands of years old, as the radiocarbon ages suggest (i.e., the traditional interpretation), then the fatty acid time series would not record the ^14^C bomb spike because the vast majority of the fatty acid biosynthesis would have predated the initiation of nuclear weapons testing^24^. If the fatty acids were exported on annual timescales, the fatty acid time series would more closely mimic the atmospheric ^14^C record in magnitude and pacing^24^. The observed fatty acid Fm time series indicate that each homologue must have at least two constituents: a fast-cycling component that incorporates bomb carbon at a rate that depends on its turnover time, and a slow-cycling component that is insensitive to the atmospheric bomb spike. The slow-cycling component dilutes the isotopic excursion recorded in the fast-cycling component with the proportions of the two components varying among homologues.

According to this observation, a two-component isotope-mixing model was constructed to quantify the calendar ages and fractional contributions of the fast- and slow-cycling components for each fatty acid homologue, expressed as

1 $${Fm}_{{\rm{F}}{\rm{A}}}={{\rm{f}}}_{{\rm{F}}{\rm{a}}{\rm{s}}{\rm{t}}}{{\rm{F}}{\rm{m}}}_{{\rm{F}}{\rm{a}}{\rm{s}}{\rm{t}}}+{{\rm{f}}}_{{\rm{S}}{\rm{l}}{\rm{o}}{\rm{w}}}{{\rm{F}}{\rm{m}}}_{{\rm{S}}{\rm{l}}{\rm{o}}{\rm{w}}}$$

and

2 $${{\rm{f}}}_{{\rm{Fast}}}+{{\rm{f}}}_{{\rm{Slow}}}=1$$

where Fm_FA_ is the measured fatty acid Fm and Fm_Fast_, Fm_Slow_, f_Fast_, and f_Slow_ are the Fm and the fractional abundances of the fast- and slow-cycling components.

To account for continental reservoirs that host OC with a smear of ages rather than a single discrete age or a combination of several discrete ages, the fast- and slow-cycling components are modeled as Gaussian age distributions that are described by the following probability distribution function:

3 $$p(t|\mu ,\sigma )=\frac{1}{\sqrt{2\pi }\sigma }\,\exp \,[\,-\,\frac{{(t-\mu )}^{2}}{2{\sigma }^{2}}]$$

where t is time, σ is the standard deviation, and μ is the center of the age distribution^22,23^. The atmospheric Fm sets the initial fatty acid radiocarbon composition, so the Fm of each component is expressed as a linear combination of sums of the atmospheric Fm (Fm_Atm_) weighed by the probability distribution function both evaluated at time *t*. Fm_Atm_ is calculated from atmospheric Δ^14^C values from Intcal13 Northern Hemisphere^25^ and Northern Hemisphere zone 3^26^ that are interpolated to yearly resolution and decayed for the time difference between time *t* and the sediment deposition year, *t*_0_. Fm_Atm_ values are set to 0 for years greater than 50,000 BP (i.e., radiocarbon-dead). The time domain is limited from t_0_ to 100,000 years BP (t_max_), such that all of the fatty acids were biosynthesized before or during the year of sediment deposition. Accordingly, Fm_Fast_ and Fm_Slow_ are calculated using the following equation:

4 $${\rm{F}}{\rm{m}}=\frac{\sum _{t={t}_{0}}^{{t}_{{\rm{m}}{\rm{a}}{\rm{x}}}}\,p(t|\mu ,\sigma )\,{{\rm{F}}{\rm{m}}}_{{\rm{A}}{\rm{t}}{\rm{m}}}(t)}{\sum _{t={t}_{0}}^{{t}_{{\rm{m}}{\rm{a}}{\rm{x}}}}\,p(t|\mu ,\sigma )}$$

where the denominator ensures that the distribution integrates to 1 after truncation of the time domain.

Fm_Slow_ and Fm_Fast_ are calculated for each sample year for a range of age distributions (i.e., μ and σ). Fm_Fast_ is calculated for 234 unique age distributions spanning average ages of 4 to 101 years. Likewise, Fm_Slow_ is calculated for 496 unique age distributions, spanning an average age range of 199 to > 50,000 years (i.e., radiocarbon-dead; Fm_Slow_ = 0). For each of the resulting 116,064 unique combinations of fast- and slow-cycling age distributions, the optimal f_Slow_ for the entire Fm_FA_ time series is determined using a standard least squares regression solver implemented in the Python package Numpy (numpy.linalg.lstsq^27^). This calculation is performed independently for each homologue, and it assumes that the fast- and slow-cycling age structures (μ and σ) and their fractional contributions are constant over the sample interval 1946–2003. Next, the calculated f_Slow_, Fm_Fast_(t), and Fm_Slow_(t) corresponding to each combination of fast- and slow-cycling distributions and for each homologue are substituted into Eqs (1) and (2) to generate synthetic fatty acid Fm time series. Finally, the root mean square error (RMSE) is calculated to determine the fit between the synthetic Fm data and the measured fatty acid data for each chain length. Fast- and slow-cycling age combinations are filtered out if the optimal f_Slow_ was determined to be less than 0 or greater than 1.

RMSE heat maps (Fig. 1 and Supplementary Fig. S9) and Table 1 show which combinations of fast- and slow-cycling age distributions best fit the measured fatty acid radiocarbon data. *n*-C_24_, *n*-C_26_, and *n*-C_28_ share similar age distributions, while the best-fitting *n*-C_30+32_ age structure is older. Compared to the long-chain fatty acids, *n*-C_16_ has younger and narrower fast- and slow-cycling age distributions. The slow-cycling component contributes centennial carbon to *n*-C_16_ and a mixture of centennial and millennial carbon to the long-chain fatty acid inventory. The slow-cycling component represents a majority of every fatty acid homologue.

**Table 1:** Summary of top eight best-fitting age distributions for individual fatty acids and combined long-chain fatty acids (Comb. *n*-C_24−32_) according to RMSE.

FA	σ_Fast_ (yrs)	σ_Slow_ (yrs)	μ_Fast_ (yrs)	μ_Slow_ (yrs)	Fast Avg. Age (yrs)	Slow Avg. Age (yrs)	f_Slow_
*n*-C_16_	5–7.5	250	0–2	0–100	4–6	199–240	0.84–0.86
*n*-C_24_	7.5	500–1250	12–15	0–1000	13–15	941–1059	0.73–0.76
*n*-C_26_	7.5–10	500–1250	15–16	0–1000	15–17	941–1028	0.80–0.84
*n*-C_28_	7.5–10	750–1500	8–9	0–1050	10–11	1167–1312	0.78–0.81
*n*-C_30+32_	12.5	750–2250	25	0–1600	26	1541–1853	0.70–0.74
Comb. *n*-C_24−32_	7.5	500–1500	15	0–1050	15	1028–1202	*n*-C_24_: 0.68–0.71
							*n*-C_26_: 0.78–0.81
							*n*-C_28_: 0.78–0.82
							*n*-C_30+32_: 0.88–0.93
							Avg. long-chain: 0.79–0.83

While each simulated fast- or slow-cycling age distribution had only a single μ and σ value, the ranges reported in the table reflect the range of individual μ and σ values that define the top eight best-fitting age distributions. The last table entry for combined long-chain fatty acids reflects the additional modeling experiment of keeping the fast and slow-cycling endmembers (μ and σ) constant for all *n*-C_24+_ fatty acids while allowing the fractional contribution to vary independently for each homologue, hence the f_slow_ values listed for each homologue under Comb. *n*-C_24−32_.

Because long-chain fatty acids share sources, continental reservoirs, and transport pathways, and the fast- and slow-cycling average ages calculated for the individual long-chain fatty acids were similar (Table 1), an additional analysis was performed to determine which age structure best represents all of the long-chain fatty acids: a combined long-chain fatty acid age structure. For this analysis, the fast- and slow-cycling endmembers were held constant (i.e., constant μ_Fast_, σ_Fast_, μ_Slow_, and σ_Slow_) for all long-chain fatty acids while allowing fractional contributions to vary for each homologue. Using this approach, a combined *n*-C_24−32_ RMSE was calculated to assess which age structures best approximate the long-chain fatty acid data on the whole. Accordingly, the eight best-fitting fast- and slow-cycling age distributions for the leaf-wax fatty acids have average ages of 15 and 1,028–1,202 calendar years, respectively, and f_Slow_ increases with increasing chain length (Fig. 1). Weighing f_Slow_ according to average fatty acid concentrations gives a leaf-wax fatty acid f_Slow_ range of 0.79 to 0.83. Despite slightly less optimal fits, the top 9–100 best-fitting age distributions for the long-chain fatty acids have similar fast and slow average ages (13–18 years and 941–1,396 years, respectively) and fractional contributions (average long-chain f_Slow_ = 0.75–0.88) (Supplementary Fig. S10, Table S9).

**Figure 2:**
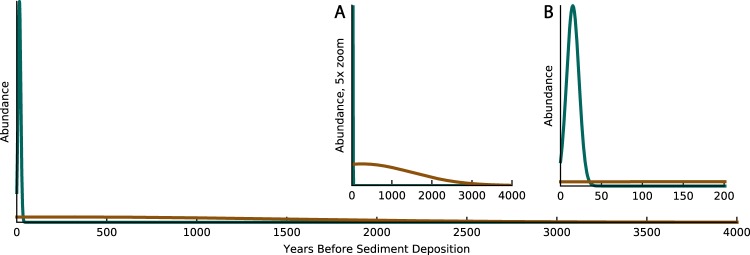
The best-fitting fast- and slow-cycling age distribution for leaf-wax fatty acids where the fast-cycling age distribution (green) and the slow-cycling age distribution (brown) are set by the following parameters: μ_Fast_ = 15 yr, σ_Fast_ = 7.5 yr, μ_Slow_ = 250 yr, and σ_Slow_ = 1,250 yr. The fast- and slow-cycling components represent 17% and 83%, respectively, of the concentration weighted leaf-wax fatty acids for this age distribution. In the model, the y-axis is related to the probability density function, but it can be conceptualized as relative fatty acid abundance that was biosynthesized in a given year and has not yet been degraded. The y-axis is rescaled in subpanel A to better show the slow-cycling distribution, and the x-axis is rescaled in subpanel B to better show the fast-cycling distribution.

The top eight best-fitting age distributions are set by μ_Fast_ = 15 years, σ_Fast_ = 7.5 years, μ_Slow_ = 0–1,050 years, and σ_Slow_ = 500–1,500 years (Table 1 and Fig. 2). The best-fitting age distributions range more in their μ_Slow_ and σ_Slow_ than in their slow-cycling average ages (1,028–1,202 years) because of an inverse relationship between μ_Slow_ and σ_Slow_ for these optimal age distributions. This inverse relationship between μ_Slow_ and σ_Slow_ means that the slow-cycling age distribution has a broader distribution if it is centered near the sediment deposition age and the distribution narrows as the center of the distribution moves further away from sediment deposition age. We argue that broader distributions centered on or near the sediment deposition age more likely represent soil profiles that accumulate modern biomass while at the same time abundances of older carbon attenuate as degradation proceeds.

Similar f_Slow_ and δ^13^C values for *n*-C_16_ and the long-chain fatty acids suggest that they are physically sourced from the same continental reservoirs. However, faster degradation rates may explain younger slow-cycling component ages for shorter-chain compounds^28,29^, particularly *n*-C_16_. Likewise, different degradation rates also explain variations in f_Slow_ for the long-chain fatty acids when the endmembers are held constant.

Our model assumes that the endmember age distributions and their fractional contributions remain constant over the sampling interval (1946–2003), despite concurrent land-use changes within the G-B catchment. While anthropogenic activity can impact sediment and OC transport, the relatively constant fatty acid δ^13^C values and homologue distributions suggests that anthropogenic activity has not significantly altered the supply of terrestrial fatty acids to the shelf canyon during the sampling interval. Moreover, the relatively constant bulk properties (TOC, Al/Si, ^87^Sr/^86^Sr, and εNd values; Supplemental Table S1) indicate relative depositional stasis over this interval. In contrast, these geochemical parameters fluctuate more significantly over thousands of years in the G-B system^15,30^, demonstrating that the system is capable of a much larger degree of variability than observed over the time interval captured in the SO188–336KL core. Although not conclusive, the observation that fatty acid and bulk OC Fm values approach pre-bomb levels in the latter part of the record supports the assumption of a stable OC source. Finally, the extensive size of the G-B rivers and Himalayas may buffer the system from anthropogenic effects over our short sampling interval.

We combine our long-chain fatty acid age structure with the bulk OC Fm to refine the previous age structure estimates of bulk OC in the G-B basin^3^ (see Supplemental Information (SI)). These results suggest that ~10–15% of the bulk OC is decadal (average age 15 years), ~47–60% is labile millennial OC (average age 1,000–1,200 years), ~23–35% is refractory OC (>15,000 years), and ~5% is petrogenic (radiocarbon-dead) (Supplementary Fig. S11). These results compare well with previous estimates of old refractory OC (>15,000 years) representing 20% of the total biospheric carbon exported from the G-B rivers^3^. Pre-aged soil OC emanating from the G-B basin thus dominates the total OC budget — a feature that has been observed in other world rivers^31^. Future research is needed to identify which combinations of microbial degradation and erosional processes generate the age structure of organic matter delivered to the Bengal Fan.

According to the fatty acid and bulk OC age distributions resolved in this study, reservoirs that host centennial and millennial carbon, such as soils, floodplains, and wetlands^24^, dominate the riverine supply of OC to Bengal Fan sediments. Successive periods of storage and remobilization of sediments pre-ages the majority of the leaf-wax fatty acids and OC delivered to the Bengal Fan^3^. Physical and climatic features such as topography, temperature, precipitation, and catchment area likely determine the endmember ages and fractional contributions, suggesting that fatty acid age structures vary spatially and temporally. While additional investigation is required before extrapolating our age structures to other localities, a significant centennial and millennial fatty acid contribution may explain the old leaf-wax radiocarbon ages observed in a wide range of environments^1,2,4,19,32^.

**Figure 3:**
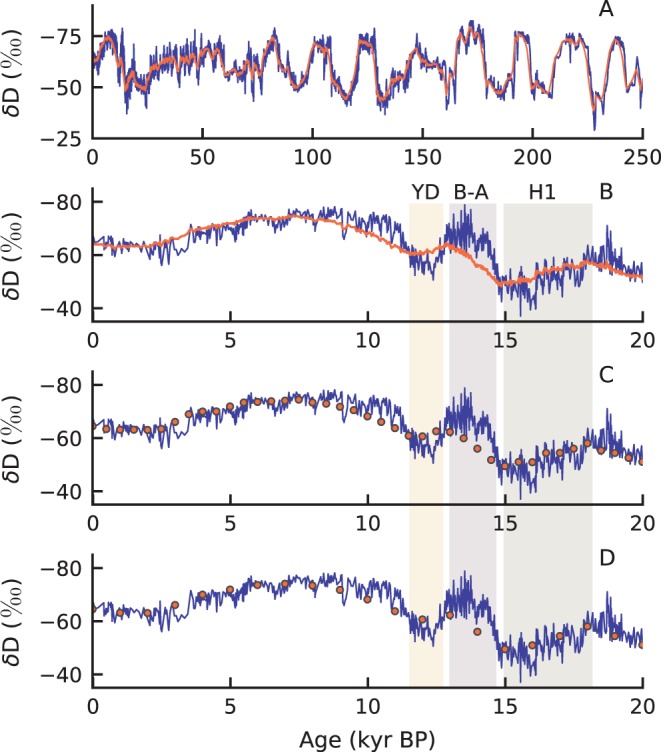
Effect of leaf-wax fatty acid age structure on paleorecords. The synthetic leaf-wax fatty acid δD record (orange) is plotted against a δD record (blue) derived from the composite δ^18^O data for Chinese cave speleothems^33,34^, spanning the last 250,000 years (panel A) and the last 20,000 years (panel B). The millennial fatty acid component damps the decadal to centennial high-frequency variability that is present in the speleothem record. The Younger Dryas (YD), Bølling-Allerød (B-A), and Heinrich 1 (H1) events are labeled. The millennial fatty acid component introduces time lags where events such as the B-A occur later in the synthetic fatty acid record than in the speleothem record. The synthetic leaf-wax fatty acid data are sampled every 500 years (panel C) and every 1,000 years (panel D).

The contribution of large amounts of pre-aged continental OC to sedimentary archives may affect paleoclimate records derived from associated biomarkers. To test the significance of this effect, we simulated a 250 kyr leaf-wax fatty acid δD sedimentary record. Accordingly, the composite δ^18^O record from Chinese cave deposits^33^ was interpolated to yearly resolution and then converted to δD using a southeast Asia meteoric water line^34^. This primary climate record was then filtered through the best-fitting long-chain fatty acid age solution (μ_Fast_ = 15 yr, σ_Fast_ = 7.5 yr, μ_Slow_ = 250 yr, σ_Slow_ = 1,250 yr, and f_Slow_ = 0.8) to generate a synthetic leaf-wax δD sedimentary record. This age structure introduces time lags across geologically rapid events, such as the Younger Dryas and Bølling-Allerød, and prevents the fatty acid record from expressing the full magnitude of the decadal to centennial high-frequency variability (Fig. 3). The time lags and high-frequency damping effects are visible if the fatty acid synthetic record is sampled on centennial or shorter timescales, but millennial or longer sampling time scales mask the incoherence.

The synthetic leaf-wax δD deviates from measured terrestrial biomarker δD records that are available from the Bay of Bengal for several reasons^30,35^. First, the Chinese caves may not record the same climate and monsoon variability that the G-B floodplain and Indo-Burma region export to the Bengal Fan and Eastern Bengal Slope, respectively. Second, the decadal to centennial high-frequency damping may not be evident in a measured biomarker δD record due to the analytical uncertainty that is associated with the δD measurement, which is on the same order of magnitude as the decadal to centennial high-frequency climate variability in the Chinese cave record^36^. Finally, the fatty acid age structure likely changes over time as climate and hydrological parameters fluctuate^22,32^ – a variable that is unaccounted for in our synthetic fatty acid δD. As such, a significant deviation between the synthetic and measured leaf-wax δD records could reflect climate-driven changes in terrestrial OC storage timescales.

The fatty acid age distributions suggest that the turnover timescales of OC stored in the G-B basin and exported to the Bengal Fan are longer than current turnover timescale estimates of total ecosystem OC (14 yr) and soil OC (25 yr)^37^. This discrepancy implies that a vast majority of the net primary production (NPP) in the G-B basin is respired on annual to decadal timescales, but the less-labile OC that escapes respiration accumulates in soils over centuries to millennia. While this OC may be less labile, functionalized lipids, including leaf-wax fatty acids, can be degraded^38^. As a result, our data imply that the G-B basin may store large quantities of degradable OC that accumulated over centuries to millennia under current and recent climate conditions. Since precipitation is a key driver of soil turnover at low latitude^39^, natural variations in the strength of the Indian monsoon likely impact the turnover timescales of this large inventory of degradable OC, suggesting that tropical terrestrial OC dynamics may represent an important global climate feedback over glacial/interglacial timescales. Rising temperatures and shifting precipitation patterns could destabilize this slow-cycling, yet potentially reactive, soil OC pool, reorganizing the balance among oxidation to the atmosphere, storage in soils, and burial in marine sediments. Our estimates of OC residence times within the G-B watershed may serve as a model to assess how soil, floodplain, and wetland response to climate and land-use change quantitatively affects the global carbon cycle.

## Methods

Decarbonated samples were analyzed for bulk ^14^C at ETH Zurich using the elemental analyzer-accelerator mass spectrometer (EA-AMS) MIcroscale CArbon DAting System (MICADAS)^40^. Samples were additionally analyzed for TOC and Al/Si elemental ratios, while a subset of samples were analyzed for Sr and Nd isotope composition. The fatty acids were isolated from sediment extracts and methylated to fatty acid methyl esters (FAMEs), which were purified and quantified. The stable carbon isotopic compositions of the FAMEs were analyzed in triplicate by gas chromatography-isotope ratio mass spectrometry (GC-IRMS). For a subset of samples, six individual saturated, straight-chain FAMEs (*n*-C_16_, *n*-C_24_, *n*-C_26_, *n*-C_28_, *n*-C_30_, and *n*-C_32_) were purified and collected for radiocarbon analysis according to the previously established preparative GC method^41^. These purified FAMEs were combusted to CO_2_ gas and analyzed at ETH Zurich using the AMS MICADAS system^42^. The ^14^C FAME data were corrected for blank contribution by isotope mass balance. Finally, the ^14^C and ^13^C FAME data were corrected for methylation. See SI for additional method details and complete data tables.

## Electronic supplementary material

Supplementary Information
