# Supplementary material for: Millennial soil retention of terrestrial organic matter deposited in the Bengal Fan
Source: Sci Rep. 2018 Aug 10;8:11997. doi: 10.1038/s41598-018-30091-8 (PMC6086914; doi:10.1038/s41598-018-30091-8)
Supplement: Supplementary file 1 — Supplementary Information [file 41598_2018_30091_MOESM1_ESM.pdf]

Supplemental Information (SI)

Millennial soil retention of terrestrial organic matter deposited in the Bengal Fan

Katherine L. French, Christopher J. Hein, Negar Haghipour, Lukas Wacker, Hermann R. Kudrass, Timothy I. Eglinton, Valier Galy

1. Methods

1.1 Sample collection

Gravity piston core SO188-336KL (18.6 m length) was retrieved by the R/V *Sonne* in *ca.* 240 m water depth along the floor of the Bengal shelf canyon "Swatch of No Ground" (SoNG; Fig. S1) in June 2006. This core was collected near the head of the SoNG, at the same location (21° 21.04' N, 89° 34.64' E) as the SO93-96KL core, which has been previously described[1-3], but capturing the intervening 13 years of sedimentation. Following collection, the core was described, logged, imaged, and stored at 4°C at the Federal Geological Survey of Germany.

A set of 37 samples was collected from the core at *ca.* 50-cm intervals. Sample thicknesses ranged from 1 to 15 cm (mean: 7.8 cm; median: 10 cm). Most samples were taken from the fine-grained top section of graded beds, which are thought to be deposited by gravity flows generated by storms along the innermost shelf. The remaining samples include a 1 cm thick core-top sample and six coarse-grained samples collected from the bottom sections of graded tempestite beds; the latter were omitted from further study. All samples were freeze-dried and homogenized, and aliquots collected for bulk inorganic and organic analyses, with the remainder (>90%) set aside for lipid extraction and compound-specific analyses.

1.2 Sediment dating

Age-depth models are based on a combination of down-core concentration variations of ^{137}Cs (Fig. S2) — an anthropogenic fallout nuclide formed during atmospheric nuclear bomb tests — and, in the upper 3.5 m, correlation between storm-driven gravity flow beds ("tempestites") and known cyclone impacts in the Bay of Bengal[1,4] (Fig. S3).

Variations in ^{137}Cs concentrations provide for dating of sediment deposited between 1954 and 1980, after which global atmospheric concentrations became negligible[5,6], with the exception of the regional effects of the Chernoybl accident. Cesium concentrations were determined from samples collected from 7–10cm thick core segments at approximately 1 m intervals down core. Measurements were made using a pure germanium gamma

spectrometer to measure gamma emissions ^{137}Cs (661 keV). Resulting ^{137}Cs activities ranged from 0.787 Bq/kg (1,850-1,857 cm) to 3.894 Bq/kg (1,600-1,610 cm), with a mean activity of 2.159 Bq/kg (Fig. S2). Peak ^{137}Cs, corresponding in the G-B drainage basin to 1965 CE[7], is observed at 16 m depth. The bottom 1.5 m of the core does not show an excess of ^{137}Cs, and thus corresponds to the period prior to the first significant occurrence of ^{137}Cs in atmospheric fallout in 1955; accretion rates during this period were linearly extrapolated to the core bottom, providing a deposition date of 1945 CE.

High-precision (sub-year) age-depth models for the upper 16 m of the core are based on correlation of seismic reflections and sedimentary units from repeat surveys and coring collection during R/V Sonne cruises 93 (February 1994; core SO93-96KL[1,3]) and 188 (June 2006; core SO188-336KL), coupled with correlation of tempestite (storm-derived) layers with the historical Bay of Bengal cyclone record for the period between February 1994 (top of core SO93-96KL) and June 2006. This latter method, first applied to core SO93-96KL[1] makes use of 2- to 15-cm thick, fining-upward, interbedded sand and silt layers found throughout the core and interpreted as tempestites. These units have distinct basal contacts, generally grade from ~80% sand and silt at their base to ~30% near the top, and lack current-induced bedding[1]. These characteristics suggest deposition by settling from suspension clouds that are mobilized and transported by tropical cyclone-induced downwelling and that, upon crossing the deeper water of the SoNG, lose their ability to transport coarser particles[1,2]. Down-core sand and silt concentrations are derived from very-high-resolution laser diffraction particle size analyzer records and positively correlated with the historical cyclone record[1]. The application of this approach to core SO188-336KL was undertaken at the University of Bremen and provided a high-resolution age model for the top 358 cm (21 years)[4,8].

1.3 Bulk measurements

The bulk-sediment weight-percent total organic carbon content (TOC) of all samples was analyzed in triplicate on an elemental analyzer coupled to a Finnigan Deltaplus isotope ratio mass spectrometer (EA/IRMS). TOC compositions were determined following fumigation acidification of powdered sample aliquots[9]. These were sealed in a vacuum desiccator with a beaker of 50 mL of 12N HCl, fumigated for 60–72 hours at 60–65°C to remove carbonates, and dried in a separate desiccator for an additional 24 hours prior to measurement. Average precision (2σ) of replicate measurements are 0.02%.

Major and trace element concentrations (used in Al/Si calculations) were determined at the Service d'Analyse des Roches et des Minéraux (SARM; Nancy, France) by IPC-AES and ICP-MS following $LiBO_2$ fusion[10] of powdered sample aliquots pre-rinsed with milli-Q water to minimize sea salt contributions.

Sr and Nd isotopic compositions were measured on powdered sample aliquots at CRPG (Nancy, France) by Thermal Ionization Mass Spectrometry following carbonate removal via leaching with 10% acetic acid[11]. Nd isotopic compositions are reported as εNd. Average uncertainties (2σ) of major/trace elemental compositions and of $^{87}Sr/^{86}Sr$ and εNd isotopic compositions are better than 2% (relative), $2x10^{-5}$, and 0.5 ε units, respectively.

1.4 Bulk Radiocarbon

Aliquots of powdered samples were weighed into silver capsules to yield between 250 and 400 μg of C. The measured TOC was used to estimate the required sample mass. The powdered sample was acidified by HCl fumigation[9] to remove inorganic carbon prior to radiocarbon analysis. The bulk radiocarbon data was acquired at ETH Zurich using the elemental analyzer-accelerator mass spectrometer (EA-AMS) MIcroscale CArbon DAting System (MICADAS)[12].

1.5 Sample preparation for molecular analysis

Sediment samples were freeze-dried and lipids were extracted from powdered sediment (~140-210 g) with a 9:1 (*v:v*) dichloromethane:methanol (DCM:MeOH) solvent mixture using a microwave-assisted reaction systems (MARS, CEMS corporation). After centrifuging, the solvent extract was decanted and collected. The sediment was solvent-rinsed and centrifuged a minimum of three times. The total lipid extract was concentrated using a Turbovap and saponified using 15 mL of 0.5 M KOH in MeOH and ~150 μL of Milli-Q water. After the solution was heated for 2 hours at 70°C, 15 mL of Milli-Q water and 0.5 g of NaCl were added to the solution. A basic lipid fraction was extracted with hexane (5 x 5 mL rinses). The remaining solution was acidified dropwise to a pH of ~2.5 using 12 N HCl. An acidic lipid fraction was extracted with 4:1 hexane:DCM (5 x 5 mL rinses). The basic and acidic lipid fractions were collected and fractionated separately. They were dried over combusted Na_2SO_4 and fractionated into compound classes by column chromatography using a stationary phase of 1 g aminopropyl-functionalized silica gel. Five fractions were eluted using 4 mL of hexane (F1; hydrocarbons), 7 mL of 4:1 hexane:DCM (F2, ketones/esters), 10 mL of 9:1 DCM:acetone (F3, alcohols and other polar lipids), 14 mL of 98:2 DCM:formic acid (F4, acids), and 17 mL of 1:1 DCM:MeOH (F5, final column flush).

The F4 fractions of the basic and acidic lipid extracts were combined into a total F4 fraction containing the fatty acids. The fatty acids were methylated with acidified MeOH of known isotopic composition by adding 15 mL of 95:5 MeOH:HCl to the dried fatty acid fraction. The samples were purged with nitrogen and heated at 70°C overnight, after which the methylation reaction was quenched with 15 mL of Milli-Q water. The fatty acid methyl esters (FAMEs) were recovered using 4:1 hexane:DCM (5 x 6 mL rinses) and dried over combusted Na_2SO_4. The FAMEs were purified further with a second aminopropyl-functionalized silica gel column. Three fractions were eluted with 4 mL of hexane (F1), 7 mL of 4:1 hexane:DCM (F2, FAMEs), and 15 mL of 1:1 DCM:MeOH (F3, column flush).

The purified FAMEs fractions were screened and quantified on a gas chromatography-flame ionization detector (GC-FID). Saturated FAMEs were further purified by silver nitrate chromatography, which removed unsaturated compounds. Three fractions were eluted from Pasteur pipettes loaded with 0.5 g of silver nitrate impregnated silica gel, where 5 mL of 95:5 hexane:DCM was used to elute F1, 18 mL of 5:1 hexane:DCM was used to elute F2 containing FAMEs, and 5 mL of 1:1 DCM:acetone was used to elute F3. The purity of the saturated FAMEs was reassessed by GC-FID prior to stable carbon isotopic analysis and preparative capillary gas chromatography (PCGC) for compound-specific radiocarbon analysis. Purified saturated FAME fractions were subsampled for stable C isotopic analyses. This study focuses on saturated, even-numbered, straight-chained fatty acids, where the n-$C_{X:0}$ fatty acid will be referred to as n-C_X (x corresponds to the carbon chain length).

1.6 Compound-specific stable carbon isotopic analysis

The stable carbon isotopic compositions of the FAMEs were acquired on an HP 6890 GC with a Gerstel CIS-4 programmable temperature vaporizing (PTV) inlet and CP-Sil 5-CB-MS column (0.25 mm i.d. x 0.25 μm phase x 60 m length) coupled via a Finnigan-MAT GCC-III (GC Combustion-III) interface[13] to a DeltaPlus gas isotope ratio mass spectrometer. The GCC-III reference gas was calibrated using a suite of nine extensively analyzed compounds injected repeatedly, resulting in an accuracy and precision averaging better than 0.3‰. Samples were analyzed in triplicate at a minimum, and the associated error represents the standard deviation from the mean.

1.7 Compound-specific radiocarbon preparation and analysis

Six individual saturated FAMEs (n-C_{16}, n-C_{24}, n-C_{26}, n-C_{28}, n-C_{30}, and n-C_{32}) were purified and collected using the PCGC method[14] using either an Agilent 7890A or HP 5890 Series II GC coupled to a Gerstel fraction

collector. The purified saturated FAMEs fractions were dissolved in either iso-octane or toluene at a concentration that yielded 0.5-1 μg on column per injection. Depending on the total FAME concentration, ~50-150 injections were performed. The compounds were eluted from the PCGC traps with 4 mL of DCM, concentrated under a nitrogen stream, and further purified by 1% deactivated silica gel column chromatography (~3 cm of gel) by eluting 4 mL of DCM. The recovery and purity was checked on a GC-FID, where yields were in the range of ~40-80% of the initial material.

If purified *n*-C_{30} and *n*-C_{32} FAME concentrations were estimated to yield C masses less than 10-15 μg, these two compounds were combined into a *n*-C_{30+32} FAME sample to increase sample size and reduce analytical uncertainty during radiocarbon analyses. The purified FAMEs were dissolved in DCM (~ 250 μL) and loaded into combusted quartz tubes. Samples were dried in each quartz tube under a high-purity nitrogen stream at 37°C, until all solvent was removed. Combusted copper oxide (~150 μg) was added to the quartz tube after solvent removal. The samples were frozen in the quartz tube in a dry ice/isopropanol slurry for several minutes before the tubes were evacuated for ~1 minute to < 30 μTorr. The dry ice/isopropanol slurry was replaced with liquid nitrogen, and the quartz tubes were flame-sealed under vacuum. The FAME samples were combusted in flame-sealed quartz tubes at 850°C for 5 hours. The following day, the quartz tubes were cracked under vacuum, releasing the evolved gas. A dry ice/isopropanol slurry was used to trap water that was produced during combustion. The sample CO_2 was trapped with liquid nitrogen and manometrically quantified before being trapped using liquid nitrogen and flame-sealed in a pyrex tube for radiocarbon analyses. Radiocarbon measurements of sample-derived CO_2 were performed at ETH Zurich between September 2015 and September 2016. The AMS MICADAS system and operation parameters used at ETH Zurich are described by Christl et al.[15].

Some samples were lost (e.g., sample tube was broken) or contaminated during the radiocarbon preparation and analysis. Carbon masses calculated on the vacuum line were compared to the GC-FID concentrations to identify contamination. Samples 292-302 cm, 905-915 cm, and 1,505-1,515 cm were the first samples that were prepared for radiocarbon and some adverse conditions were noted during their preparation. In the case of sample 905-915 cm, a capillary broke in the preparative fraction collector during the PCGC preparation, which likely led to the observed low sample recoveries, and these samples had higher carbon masses on the vacuum line than expected compared to the GC-FID quantifications. Additional peaks were noted in the GC-FID chromatograms of the PCGC isolated fatty acids from 292-302 cm and 1,505-1,515 cm. These peaks likely contributed to the larger carbon masses on the vacuum line than estimated from the fatty acid quantification

on the GC-FID. These three samples do not differ from the remaining samples in the following characteristics: fatty acid distributions, total fatty acid concentrations, TOC values, ^{137}Cs values, bulk organic ^{14}C, Al/Si ratios, fatty acid $\delta^{13}C$ values, or mean grain size. Therefore, it was concluded that these samples were indeed contaminated, so they were not included in the fatty acid age distribution modeling.

2. Radiocarbon notation

Radiocarbon data are presented and discussed in terms of fraction modern (Fm):

$$Fm = {}^{14/12}C_{Sample}/{}^{14/12}C_{Modern} \quad \text{(S1)}$$

where a ^{13}C value of -25‰ is used to correct the Fm for mass-dependent fractionation. Fm is used in isotope mass-balance equations where it mixes linearly. Radiocarbon age can be calculated from Fm according to the following equation:

$$\text{Radiocarbon Age} = -8033*\ln(Fm) \quad \text{(S2)}$$

In the model, all years are on the Before Present (BP) time scale where 1950 is 0 BP following radiocarbon convention. Therefore, sample years after 1950 CE are negative on the BP time scale within the model. However, samples years are discussed on the CE calendar system, and simulation inputs and outputs are in the units of calendar years.

3. Blank Determination for Compound-Specific ^{14}C analysis

It is assumed that the preparative GC and vacuum line preparation are the primary sources of ^{14}C contamination. The purification steps prior to the PCGC isolation are not considered in the following blank assessment. In order to characterize the magnitude and isotopic composition of the blank contribution to samples during the PCGC and vacuum line preparation, two solvent blank PCGC analyses were performed where pure solvent, rather than sample, was injected. These experiments were performed under the same analytical conditions described above for the fatty acid samples, and 110 and 80 injections were completed for the first and second experiment, respectively. The first three traps were opened within a minute of the retention time corresponding to when n-C_{18} typically elutes. Likewise, the final three traps were opened within a minute of when n-C_{30} typically elutes. The first three traps were eluted into 4 mL vials that were spiked with 10, 25, and 40 μg of a modern n-C_{18} FAME standard (Fm = 1.1124). The last three traps were eluted into 4 mL vials that were spiked with 10, 25, and 40 μg of a dead n-C_{30} FAME standard (Fm = 0.0). The FAME standards and isotopic measurements were provided courtesy of Li Xu (NOSAMS, Woods Hole, MA, USA). After this point, the blank samples were prepared according to the same sample protocol described for the sample fatty acid radiocarbon analyses. Radiocarbon measurements of blank-derived CO_2 were performed at ETH Zurich

between September 2015 and March 2016. Some blank traps had an anomalous degree of contamination, so they were excluded from the blank calculations. These samples had much high carbon masses than were expected based on the spike concentrations. This contamination was likely introduced through leaks during vacuum line preparation, as indicated by the detection of non-condensable gases, or failing to successfully evacuate and flame-seal sample requiring that the sample be transferred and re-prepared for vacuum line preparation.

The two different blank experiments with different number of injections yielded similar results (Table S6). Therefore, these two datasets were combined to determine the blank mass and isotopic composition. Following the approach described by Santos et al.[16] and Shah Walter et al.[17], the mass of the blank contribution was determined by splitting the blank into modern and dead components. The magnitude and isotopic composition of the blank can then be described according to the following mass balance equations:

$$m_{Meas}*Fm_{Meas} = m_{Std}*Fm_{Std} + m_{B\_Dead}*Fm_{B\_Dead} + m_{B\_Mod}*Fm_{B\_Mod} \quad \text{(S3)}$$

and

$$m_{Meas} = m_{Std} + m_{B\_Dead} + m_{B\_Mod} \quad \text{(S4)}$$

where m_{Meas} is the measured carbon mass, Fm_{Meas} is the measured Fm, m_{Std} is the mass of standard added, Fm_{Std} is the known Fm of the standard, and the sum of the mass of the dead blank component (m_{B_Dead}) and the mass of the modern blank component (m_{B_Mod}) equal the total mass of the process blank (m_{PB}). The Fm of the dead and modern components are assigned 0.0 and 1.0, respectively. The mass of the modern blank component was calculated using the dead n-C_{30} FAME standard, where equation S3 simplifies to

$$Fm_{Meas} = m_{B\_Mod} / m_{Meas} \quad \text{(S5)}$$

which can be plotted according to $y = mx+b$ where $y = Fm_{Meas}$, $x = 1/m_{Meas}$, slope $m = m_{B\_Mod}$, and the y intercept = Fm_{B_Dead}, which should be close to the known value of 0 (Fig. S4). An uncertainty-weighted Model II regression was calculated using the lsqfitma.py Python routine (https://github.com/pyoceans/python-oceans//blob/master/oceans/ff_tools/teaching.py). The regression parameters are listed in Table S7 ($R^2 = 0.82$, the slope = 0.16 +/- 0.07, and the y intercept is 0.0063 +/- 0.0040). The regression has an R^2 of 0.82, and the y intercept is within 2 standard deviations of the known n-C_{30} FAME standard value of 0.0. According to this method, the m_{B_Mod} = 0.2 +/- 0.1 μg.

Similarly, the magnitude of the dead blank component was calculated graphically using the modern n-C_{18} FAME data (Fig. S4) and the following rearranged equation written as a function of $1/m_{Meas}$:

$$Fm_{Meas} = ((m_{B\_Mod} - (m_{B\_Dead} + m_{B\_Mod})* Fm_{Std}) / m_{Meas}) + Fm_{Std} \quad \text{(S6)}$$

The same uncertainty-weighted Model II regression was applied to calculate m_{B_Dead} from the slope substituting the value for m_{B_Mod} calculated in the previous regression and the known value of the modern n-C_{18} FAME standard for Fm_{Std}. The regression parameters are listed in Table S7 (R^2 = 0.96, the slope = -2.41 +/- 0.48, and the y intercept is 1.1437 +/- 0.0186). The regression has an R^2 of 0.96, and the y intercept within 2 standard deviations of the known n-C_{18} FAME standard value of 1.1124. This approach yields an m_{B_Dead} of 2.1 +/- 0.4 μg. Combining the modern and dead blank components and propagating the errors according to

$$m_{PB}*Fm_{PB} = m_{B\_Dead}*Fm_{B\_Dead} + m_{B\_Mod}*Fm_{B\_Mod} \quad \text{(S7)}$$

yields a combined blank mass m_{PB} of 2.2 +/- 0.4 μg and a combined blank fraction modern Fm_{PB} of 0.07 +/- 0.03 (Table S8).

4. Data Reduction

3.1 Blank and methylation correction for fatty acid radiocarbon data

The measured FAMEs fraction modern data are corrected for blank contribution during the PCGC and vacuum line preparation according to the mass balance equations:

$$m_{Meas}*Fm_{Meas} = m_T*Fm_T + m_{PB}*Fm_{PB} \quad \text{(S8)}$$

and

$$m_{Meas} = m_T + m_{PB} \quad \text{(S9)}$$

where m_T and Fm_T are the true sample mass and fraction modern, respectively, without blank carbon contribution.

The blank-corrected Fm data are further corrected for a single carbon addition during the methylation step according to the following equation:

$$Fm_{T,\,FA} = ((n + 1)*Fm_{T,FAME} – Fm_{MeOH}) / n \quad \text{(S10)}$$

where $Fm_{T,\,FA}$ is the methylation and blank-corrected fatty acid Fm, $Fm_{T,FAME}$ is the blank-corrected FAME Fm, Fm_{MeOH} is the Fm of the MeOH used during the fatty acid methylation, and n equals the purified fatty acid chain length. In the case where n-C_{30} and n-C_{32} were combined into n-C_{30+32}, average chain length (ACL) is substituted for n in equation S10, where ACL is determined according to a concentration weighted average:

$$ACL = ((30*[C_{30}]) + (32*[C_{32}])) / [C_{30+32}] \quad \text{(S11)}$$

The reported error for the corrected fatty acid Fm represents the propagated 1σ error through blank and methylation corrections. The carbon masses measured on the vacuum line were assigned an error of +/- 5%.

3.2 Methylation correction for fatty acid ^{13}C data

The fatty acid stable carbon isotopic data are also corrected for a single carbon addition during methylation according to the following mass balance equation:

$$\delta^{13}C_{FA} = ((n + 1) * \delta^{13}C_{Meas, FAME} - \delta^{13}C_{MeOH}) / n \quad \text{(S12)}$$

where $\delta^{13}C_{MeOH}$ is the stable carbon isotopic composition of the MeOH used for methylation, $\delta^{13}C_{Meas, FAME}$ is the measured FAME stable carbon isotopic composition, *n* equals the purified fatty acid chain length, and $\delta^{13}C_{FA}$ is the methylation corrected fatty acid stable carbon isotopic composition. The reported error reflects the propagated 1σ error from the analytical error and the error associated with the $\delta^{13}C_{MeOH}$.

5. Numerical simulations of fatty acid age structure

The incorporation of bomb carbon into the fatty acids demonstrates that measured fatty acid radiocarbon ages, which are older than the initiation of nuclear weapons testing, mask a mixture of an old component that is relatively insensitive to the atmospheric bomb spike and a fast-cycling component that incorporates bomb carbon. Accordingly, a two-component isotope-mixing model was constructed to quantify the ages and fractional contributions of the fast- and slow-cycling components, which can be expressed as

$$Fm_{FA} = f_{Fast} * Fm_{Fast} + f_{Slow} * Fm_{Slow} \quad \text{(S13)}$$

and

$$f_{Fast} + f_{Slow} = 1 \quad \text{(S14)}$$

where Fm_{FA} is the measured fatty acid Fm, Fm_{Fast} and Fm_{Slow} are the Fm of the fast- and slow-cycling components, and f_{Fast} and f_{Slow} are the fractional abundances of the fast- and slow-cycling components.

Rather than assigning a discrete age to each of the components, normal (Gaussian) age distributions were used to characterize the fast- and slow-cycling components[18,19]. This approach takes into account that continental reservoirs host organic matter with a smear of ages rather than a single discrete age or a combination of several discrete ages. The age distributions are described by the following probability distribution function:

$$p(t|\ \mu,\sigma) = (1/\ \sigma(2\pi)^{0.5}) * \exp(-(t-\mu)^2/2\sigma^2) \quad \text{(S15)}$$

where σ is the standard deviation or width of the distribution and μ is the mean or center of the age distribution. The Fm of the two components are expressed as a linear combination of sums of the atmospheric Fm (Fm_{Atm}) weighted by the probability distribution function both evaluated at time *t*:

$$Fm_{Fast} = {}^{\infty}\sum_{t=t0} p_{Fast}(t|\ \mu_{Fast},\sigma_{Fast}) * Fm_{Atm}(t) \quad \text{(S16)}$$

and

$$Fm_{Slow} = {}^{\infty}\sum_{t=t0} p_{Slow}(t|\ \mu_{Slow},\sigma_{Slow}) * Fm_{Atm}(t). \quad \text{(S17)}$$

The time domain is limited from the sediment deposition year t_0 to 100,000 years BP (t_{max}). This truncates the age distributions at the sediment deposition year (t_0) such that all of the fatty acids were biosynthesized before or during the year of sediment deposition. Importantly, organic matter older than 50,000 BP is considered radiocarbon dead, so organic matter older than 50,000 BP is indistinguishable by radiocarbon. In order to account for truncation, the areas of the normal distributions are normalized so that they integrate to 1:

$$Fm_{Fast} = \left(\sum_{t=t0}^{tmax} p_{Fast}(t|\ \mu_{Fast},\ \sigma_{Fast})*Fm_{Atm}(t)\right)/ \sum_{t=t0}^{tmax} p_{Fast}(t|\ \mu_{Fast},\ \sigma_{Fast}) \quad \text{(S18)}$$

and

$$Fm_{Slow} = \left(\sum_{t=t0}^{tmax} p_{Slow}(t|\ \mu_{Slow},\ \sigma_{Slow})*Fm_{Atm}(t)\right)/ \sum_{t=t0}^{tmax} p_{Slow}(t|\ \mu_{Slow},\ \sigma_{Slow}). \quad \text{(S19)}$$

As a result of truncation, μ deviates from the average age of the distribution, so it primarily represents the age offset of the distribution center relative to the sediment deposition year t_0.

Atmospheric radiocarbon composition, which sets the original fatty acid radiocarbon composition, varies over the defined time domain due to natural variability as well as inputs from nuclear weapons testing. Atmospheric Fm values are calculated from atmospheric $\Delta^{14}C$ values and decayed for the time difference between time t and t_0 according to the following equation:

$$Fm_{Atm}(t)= (1+ \Delta^{14}C_{Atm}(t)/1{,}000)*\exp(-(t-t_0)/8{,}267) \quad \text{(S20)}$$

Atmospheric Fm values from 0 to 50,000 years BP are calculated from the Intcal13 Northern Hemisphere atmospheric $\Delta^{14}C$ data and interpolated to yearly resolution[20]. Atmospheric Fm values from -60 to 0 years BP are calculated from the Northern Hemisphere zone 3 atmospheric $\Delta^{14}C$ data, which is a spatial region that covers the sample locality and the G-B river catchment area[7]. Atmospheric Fm values are set to 0 for years greater than 50,000 BP (i.e., radiocarbon dead).

Fm_{Slow} and Fm_{Fast} are calculated for each sample year according to equations S18-20 for a range of age distributions. Thirty-nine values of σ_{Fast} are evaluated, ranging from 5 to 100 years, spaced at 2.5-year increments. Likewise, σ_{Slow} is assigned 45 values starting at 250 years and spaced at 250-year increments to 5,000 years, then spaced at 500-year increments from 5,000 to 10,000 years, then spaced at 1,000-year increments from 10,000 to 25,000 years. These σ ranges allow us to evaluate a range of narrow to broad distributions. Following the approach by Fornace[18], μ_{Slow} must be within two standard deviations of the sediment year t_0 (i.e., $0 \leq \mu \leq 2\sigma$). This constraint accommodates continental reservoirs that store millennial carbon but also host decadal and centennial carbon, such as soils, floodplains, and wetlands. In other words, this constraint requires some overlap between the slow-cycling component and more recent carbon, such that the slow-cycling component does not derive from an isolated pool of purely old carbon. For every σ_{Slow}, μ_{Slow}

was assigned eleven values evenly spaced from 0 to $2\sigma_{Slow}$. Likewise, for $\sigma_{Fast} \leq 25$ years, μ_{Fast} is assigned six equally spaced values between 0 and $2\sigma_{Fast}$. For $\sigma_{Fast} > 25$ years, μ_{Fast} is allowed to range from 0 to 50 at increments of 10 years. Combining σ_{Fast} and μ_{Fast} gives 234 unique age distributions spanning average ages of 4 to 101 years for the fast-cycling component. Combining σ_{Slow} and μ_{Slow}, gives 495 unique slow-cycling age distributions. A pure radiocarbon dead age distribution is also considered for the slow-cycling component to represent paleo-soil or bedrock fatty acid contribution. In this case, Fm_{Slow} is set to 0. These different possible slow-cycling age distributions span an average age range from 199 to >50,000 years. Coupling each fast- and slow-cycling age distribution yields a total of 116,064 combinations of fast- and slow-cycling age distributions.

In order to determine which age distribution combinations best approximate the measured fatty acid Fm, it is assumed that the fast- and slow-cycling age structures and their fractional contributions are constant over the sample interval 1946-2003. For each age distribution combination and their corresponding Fm_{Fast} and Fm_{Slow}, the optimal f_{Slow} across the sampling interval is calculated according to least squares regression for each chain length. Equations S13 and S14 are combined to form

$$Fm_{FA}(t_i) - Fm_{Fast}(t_i) = f_{Slow} * (Fm_{Slow}(t_i) - Fm_{Fast}(t_i)) \quad \text{(S21)}$$

at sample year t_i. This equation is in the form y=mx+c, where $y = Fm_{FA}(t_i) - Fm_{Fast}(t_i)$, $m = f_{Slow}$, $x = Fm_{Slow}(t_i) - Fm_{Fast}(t_i)$, and c = 0. Using equation S21 for a given chain length, we obtain an overdetermined system of equations; each equation corresponding to a sample year t_i and the only unknown being f_{Slow}. The optimal f_{Slow} that minimizes squared error across all sample years is determined using a standard least squares regression solver implemented in the Python package Numpy (numpy.linalg.lstsq[21]).

For each chain length, the calculated f_{Slow}, $Fm_{Fast}(t)$, and $Fm_{Slow}(t)$ corresponding to each combination of fast- and slow-cycling ages are substituted into equations S13 and S14 to generate synthetic Fm time series. Finally, the root mean squared error (RMSE) is calculated to determine the fit between the synthetic Fm data and the measured fatty acid data for each chain length. In addition to calculating RMSE for each individual fatty acid, a combined C_{24-32} RMSE is calculated for each age structure combination in order to identify age structures that best approximate the measured long-chain fatty acid data on a whole. Fast- and slow-cycling age combinations are filtered out if the optimal f_{Slow} was determined to be less than 0 or greater than 1.

The model allows the slow-cycling distribution to overlap with the fast-cycling distribution. Arguably, the intersecting fraction of the slow-cycling component could belong to the fast-cycling component, meriting a correction of f_{Slow} to smaller values. However, this effect is small for best-fitting solutions where only a small

percent of the slow-cycling component overlaps the fast-cycling component (< 3% for long-chain fatty acids and 4-8% for n-C_{16}).

6. Geochemistry and modeling results

The bulk geochemistry shows little variability over the sample set (Table S1). The total organic carbon (TOC; range: 0.39 - 0.61%) is positively correlated with Al/Si ratios (range: 0.32 - 0.44) (Fig. S5), which suggests similar particle loading as observed in the G-B river system[22-24]. Including the SO188-336KL core-top, seven core-top samples from the Swatch of No Ground (SoNG), Bengal shelf, and active channel-levee system have bulk OC ^{14}C contents and $\delta^{13}C$ values that are compatible with the signature of sediments from the modern Lower Meghna River[25] (the confluence of the Ganges, Brahmaputra, and Meghna rivers). Specifically, the petrogenic carbon concentrations and biospheric OC average residence times from the core-top sediments are comparable to Lower Meghna sediments. The $^{87}Sr/^{86}Sr$ ratios (range: 0.7430 - 0.7484) and the εNd values (range: -14.1 to -14.9) from the SO188-336KL sample subset are characteristic of G-B river sediments[26]. The bulk geochemical data imply that the G-B rivers supply the fine-grained sediments that are deposited at the head of the SoNG, and the sediment source has not changed over the sampling interval[11,26-28].

The average fatty acid concentrations for the even-numbered fatty acid homologues are plotted in Fig. S6, where a bimodal distribution centered at n-C_{16} and n-C_{28} is evident. The fatty acid $\delta^{13}C$ results are plotted as a function of sample year and carbon number in Fig. S7. The measured bulk OC and fatty acid radiocarbon data are plotted as a function of sample year in comparison to the atmospheric radiocarbon record in Fig. S8. RMSE heat maps for each individual fatty acid homologue and the combined C_{24-32} RMSE heat map over the entire solution space tested in the model (slow-cycling average age up to >50,000) is shown in Fig. S9. Synthetic fatty acid time series are plotted against the measured data for less optimal age distribution solutions in Fig. S10.

7. Bulk organic carbon age distribution

Down-core bulk OC values record a muted bomb spike that is offset below the weighted average long-chain fatty acid Fm (Fig. S8). An additional old OC component, devoid of n-C_{16} and long-chain fatty acids, explains the translation of bulk OC Fm to lower values. Previously, terrestrial OC in the G-B floodplain and delta was apportioned into ~5% petrogenic carbon, 10-29% refractory biospheric carbon exceeding an average age of 15,000 years, and 66-85% labile biospheric carbon[29,30]. By construction, the refractory biospheric carbon does not contain long-chain fatty acids because the average age of 15,000 years was derived by extrapolating to 0

μg/g *n*-C_{24+} fatty acid concentration[29], and unlike *n*-alkanes, petrogenic sources do not contribute to the fatty acid inventory[31,32]. Indeed, our model results suggest that long-chain fatty acids are absent in terrestrial OC exceeding 15,000 years because nearly 100% of the fatty acid inventory is deposited in the Bengal sediments within 0 to 2,500-5,000 years of biosynthesis, depending on the best-fitting age distributions.

In order to characterize the age structure of the bulk OC, a mass balance is written in terms of a labile biospheric component (Lb) that contains fatty acids, a refractory biospheric component (Rf), and a petrogenic component (Pt):

$$Fm_{Bulk} = f_{Lb}Fm_{Lb} + f_{Rf}Fm_{Rf} + f_{Pt}Fm_{Pt} \quad \text{(S22)}$$

and

$$f_{Lb} + f_{Rf} + f_{Pt} = 1 \quad \text{(S23)}$$

Based on previous estimates[29,30], Fm_{Pt} and f_{Pt} are assigned 0 and 5%, respectively, which simplifies the mass balance to

$$f_{Lb} = (Fm_{Bulk} – 0.95*Fm_{Rf})/(Fm_{Lb} – Fm_{Rf}) \quad \text{(S24)}$$

Assuming that marine contribution to the Bengal Fan bulk OC is negligible[33] and the long-chain fatty acids are representative of the labile terrestrial biospheric OC, the average long-chain fatty acid Fm (Fm_{C24+FA}) is substituted for Fm_{Lb}, and the Fm_{Slow} time series calculated in the fatty acid mixing model simulations are substituted for Fm_{Rf}. Next, f_{Lb} is calculated according to least squares regression. Several values of Fm_{C24+FA} and Fm_{Bulk} were interpolated to achieve equivalent time resolution between datasets.

Analogous to the fatty acid mixing model, a synthetic bulk time series is calculated using f_{Lb} for each refractory distribution. Refractory average ages of ≤ 7,500 years require larger f_{Rf} values that flatten the bomb spike signature, preventing the synthetic curve from reaching the maximum values observed in the bulk OC (Fig. S11). Refractory average ages of ≥ 7,500 years approach the full magnitude of the bomb spike recorded in the bulk OC data. If the refractory average age range is conservatively allowed to range from 7,500 to > 50,000 years (i.e., radiocarbon-dead), then f_{Lb} ranges from 0.45 to 0.72 and f_{Rf} ranges from 0.23 to 0.50. Increasing the refractory average age minimum to 15,000 years[29] narrows the range of f_{Lb} to 0.60-0.72 and the range of f_{Rf} to 0.23-0.35. Using these latter fractional abundance ranges, we propose an idealized age structure of bulk OC hosted in recent Bengal sediments where the labile biospheric component is subdivided into a decadal and millennial component based on the weighted average long chain fatty acid modeling results (see main text for results).

References

1 Kudrass, H., Michels, K. H., Wiedicke, M. & Suckow, A. Cyclones and tides as feeders of a submarine canyon off Bangladesh. *Geology* **26**, 715-718 (1998).

2 Michels, K. H., Suckow, A., Breitzke, M., Kudrass, H. R. & Kottke, B. Sediment transport in the shelf canyon "Swatch of No Ground" (Bay of Bengal). *Deep Sea Research Part II: Topical Studies in Oceanography* **50**, 1003-1022, doi:10.1016/s0967-0645(02)00617-3 (2003).

3 Michels, K. H., Kudrass, H. R., C.Hubscher, Suckow, A. & Wiedicke, M. The submarine delta of the Ganges–Brahmaputra: cyclone-dominated sedimentation patterns. *Marine Geology* **149**, 133-154 (1998).

4 Kudrass, H., Machalett, B., Palamenghi, L. & Meyer, I. in *EGU General Assembly Conference Abstracts* Vol. 19 10005 (2017).

5 Pennington, W., Tutin, T. G., Cambray, R. S. & Fisher, E. M. Observations on Lake Sediments using Fallout ^{137}Cs as a Tracer. *Nature* **242**, 324-326 (1973).

6 Walling, D. & Quine, T. The use of caesium-137 measurements in soil erosion surveys. *Erosion and sediment transport monitoring programmes in river basins* **210**, 143-152 (1992).

7 Hua, Q., Barbetti, M. & Rakowski, A. Z. Atmospheric Radiocarbon for the Period 1950–2010. *Radiocarbon* **55**, 2059-2072, doi:10.2458/azu_js_rc.v55i2.16177 (2013).

8 Kudrass, H., Machalett, B., Palamenghi, L. & Meyer, I. Frequency and intensity changes of tropical cyclones during the last century recorded in a canyon of the northen Bay of Bengal. (in preparation).

9 Whiteside, J. H. *et al.* Pangean great lake paleoecology on the cusp of the end-Triassic extinction. *Palaeogeography, Palaeoclimatology, Palaeoecology* **301**, 1-17, doi:10.1016/j.palaeo.2010.11.025 (2011).

10 Carignan, J., Hild, P., Mevelle, G., Morel, J. & Yeghicheyan, D. Routine analyses of trace elements in geological samples using flow injection and low pressure on-line liquid chromatography coupled to ICP-MS: a study of geochemical reference materials BR, DR-N, UB-N, AN-G and GH. *Geostandards and Geoanalytical Research* **25**, 187-198 (2001).

11 Pierson-Wickmann, A.-C., Reisberg, L., France-Lanord, C. & Kudrass, H. R. Os-Sr-Nd results from sediments in the Bay of Bengal: Implications for sediment transport and the marine Os record. *Paleoceanography* **16**, 435-444, doi:10.1029/2000pa000532 (2001).

12 Ruff, M. *et al.* On-line radiocarbon measurements of small samples using elemental analyzer and MICADAS gas ion source. *Radiocarbon* **52**, 1645–1656, doi:10.7892/boris.5178 (2010).

13 Goodman, K. J. Hardware modifications to an isotope ratio mass spectrometer continuous-flow interface yielding improved signal, resolution, and maintenance. *Analytical chemistry* **70**, 833-837 (1998).

14 Eglinton, T. I., Aluwihare, L. I., Bauer, J. E., Druffel, E. R. M. & McNichol, A. P. Gas Chromatographic Isolation of Individual Compounds from Complex Matrices for Radiocarbon Dating. *Anal Chem* **68**, 904-912 (1996).

15 Christl, M. *et al.* The ETH Zurich AMS facilities: Performance parameters and reference materials. *Nuclear Instruments and Methods in Physics Research Section B: Beam Interactions with Materials and Atoms* **294**, 29-38, doi:10.1016/j.nimb.2012.03.004 (2013).

16 Santos, G. M. *et al.* Blank Assessment for Ultra-Small Radiocarbon Samples: Chemical Extraction and Separation Versus AMS. *Radiocarbon* **52**, 1322-1335, doi:10.1017/s0033822200046415 (2016).

17 Shah Walter, S. R. *et al.* Ultra-Small Graphitization Reactors for Ultra-Microscale 14C Analysis at the National Ocean Sciences Accelerator Mass Spectrometry (NOSAMS) Facility. *Radiocarbon* **57**, 109-122, doi:10.2458/azu_rc.57.18118 (2016).

18 Fornace, K. L. *Late Quaternary climate variability and terrestrial carbon cycling in tropical South America* Ph.D. thesis, MIT, (2016).

19 Douglas, P. M. J. *et al.* Pre-aged plant waxes in tropical lake sediments and their influence on the chronology of molecular paleoclimate proxy records. *Geochimica et Cosmochimica Acta* **141**, 346-364, doi:10.1016/j.gca.2014.06.030 (2014).

20 Reimer, P. J. *et al.* INTCAL13 and MARINE13 radiocarbon age calibration curves 0–50,000 years CAL BP. *Radiocarbon* **55**, 1869-1887 (2013).

21 van der Walt, S., Colbert, S. C. & Varoquaux, G. The NumPy Array: A Structure for Efficient Numerical Computation. *Computing in Science and Engineering* **13**, 22-30, doi:10.1109/mcse.2011.37 (2011).

22 Galy, V. *et al.* Efficient organic carbon burial in the Bengal fan sustained by the Himalayan erosional system. *Nature* **450**, 407-410, doi:10.1038/nature06273 (2007).

23 Galy, V., Eglinton, T., France-Lanord, C. & Sylva, S. The provenance of vegetation and environmental signatures encoded in vascular plant biomarkers carried by the Ganges–Brahmaputra rivers. *Earth and Planetary Science Letters* **304**, 1-12, doi:10.1016/j.epsl.2011.02.003 (2011).

24 Galy, V., France-Lanord, C. & Lartiges, B. Loading and fate of particulate organic carbon from the Himalaya to the Ganga–Brahmaputra delta. *Geochimica et Cosmochimica Acta* **72**, 1767-1787, doi:10.1016/j.gca.2008.01.027 (2008).

25 Galy, V., Hein, C., France-Lanord, C. & Eglinton, T. in *Biogeochemical Dynamics at Major River-Coastal Interfaces: Linkages with Global Change* (eds T. Bianchi, M. Allison, & W.-J. Cai) Ch. 13, 353-372 (Cambridge University Press, 2014).

26 Galy, V., France-Lanord, C., Peucker-Ehrenbrink, B. & Huyghe, P. Sr–Nd–Os evidence for a stable erosion regime in the Himalaya during the past 12Myr. *Earth and Planetary Science Letters* **290**, 474-480, doi:10.1016/j.epsl.2010.01.004 (2010).

27 Lupker, M., France-Lanord, C., Galy, V., Lavé, J. & Kudrass, H. Increasing chemical weathering in the Himalayan system since the Last Glacial Maximum. *Earth and Planetary Science Letters* **365**, 243-252, doi:10.1016/j.epsl.2013.01.038 (2013).

28 Hein, C. J. *et al.* Post-glacial climate forcing of surface processes in the Ganges–Brahmaputra river basin and implications for carbon sequestration. *Earth and Planetary Science Letters* **478**, 89-101, doi:10.1016/j.epsl.2017.08.013 (2017).

29 Galy, V. & Eglinton, T. Protracted storage of biospheric carbon in the Ganges–Brahmaputra basin. *Nature Geoscience* **4**, 843-847, doi:10.1038/ngeo1293 (2011).

30 Galy, V., Beyssac, O., France-Lanord, C. & Eglinton, T. Recycling of Graphite During Himalayan Erosion: A Geological Stabilization of Carbon in the Crust. *Science* **322**, 943-945 (2008).

31 Kusch, S., Rethemeyer, J., Schefuß, E. & Mollenhauer, G. Controls on the age of vascular plant biomarkers in Black Sea sediments. *Geochimica et Cosmochimica Acta* **74**, 7031-7047, doi:10.1016/j.gca.2010.09.005 (2010).

32 Tao, S., Eglinton, T. I., Montluçon, D. B., McIntyre, C. & Zhao, M. Pre-aged soil organic carbon as a major component of the Yellow River suspended load: Regional significance and global relevance. *Earth and Planetary Science Letters* **414**, 77-86, doi:10.1016/j.epsl.2015.01.004 (2015).

33 Hein, C. J., Galy, V., France-Lanord, C., Galy, A. & Kudrass, H. Post-Glacial Climate Forcing of Surface Processes in the Ganges-Brahmaputra River Basin and Implications for the Global Carbon Cycle. *Earth and Planetary Science Letters* (In Press).

34 Blaauw, M. Methods and code for 'classical' age-modelling of radiocarbon sequences. *Quaternary Geochronology* **5**, 512-518, doi:10.1016/j.quageo.2010.01.002 (2010).

Figures and Tables

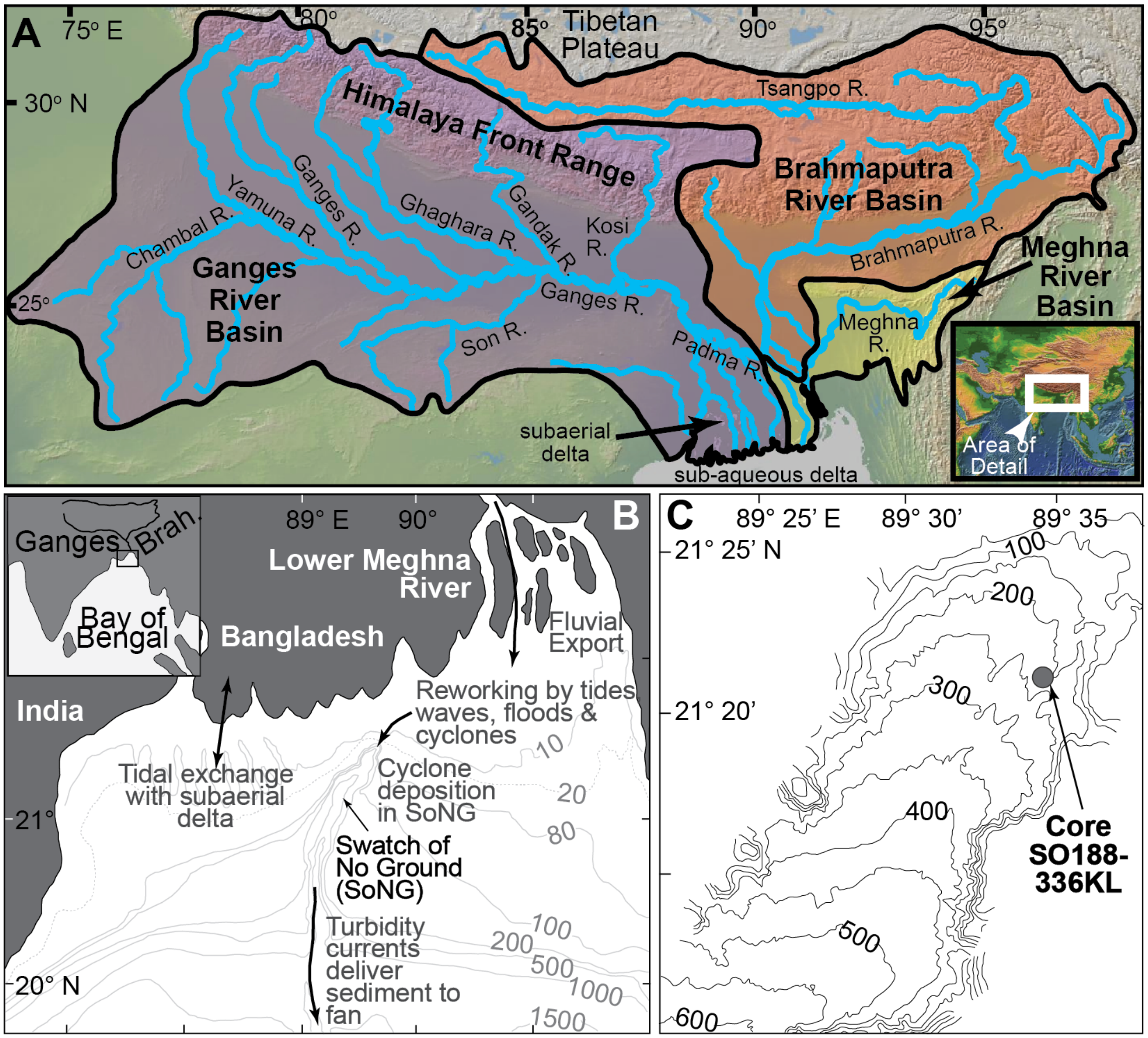

Figure S1. Site of core SO188-336KL at the head of the Bengal Shelf Swatch of No Ground (SoNG). A) The drainage basin and tributaries of the G-B rivers (modified from Hein et al.[28]). B) Sediment transport pathways and processes from the mouth of the G-B rivers across the Bengal Shelf[1,25]. C) Detailed bathymetry of upper SoNG showing core recovery location[2]. All depth contours are in meters.

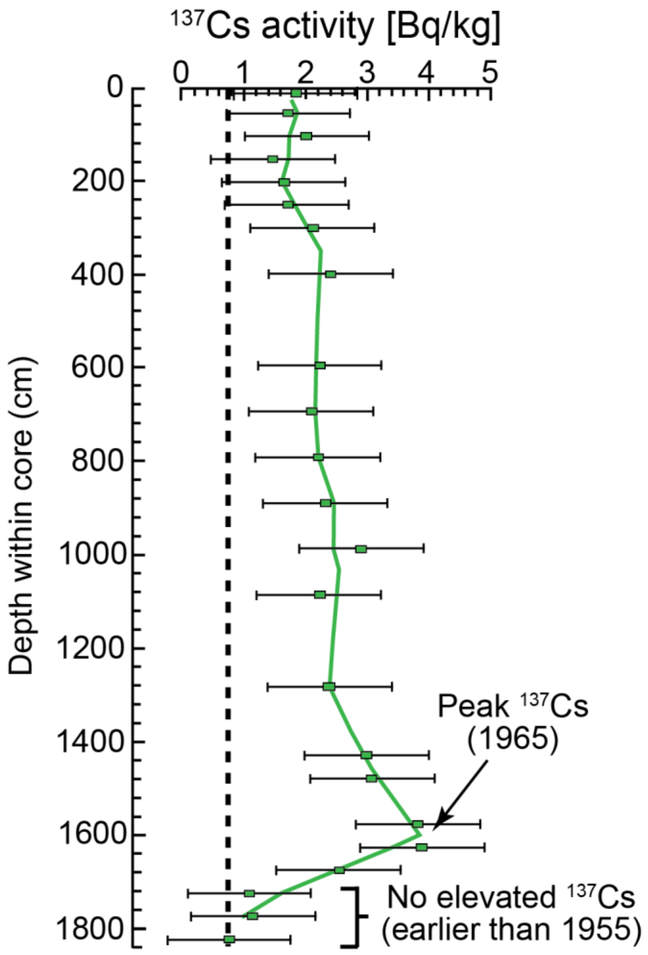

Figure S2. Down-core ^{137}Cs profile (raw data points and 3-point moving average line) from core SO188-336KL. The "bomb spike"—an anthropogenic pulse of ^{137}Cs generated by atmospheric testing of atomic weapons—is clearly visible. The dashed line represents the measured ^{137}Cs background. Note that the bottom ca. 1.5 m of the core has no elevated ^{137}Cs levels and therefore corresponds to the years prior to 1955. The peak of the bomb spike at *ca.* 16 m corresponds to the mid-1960s.

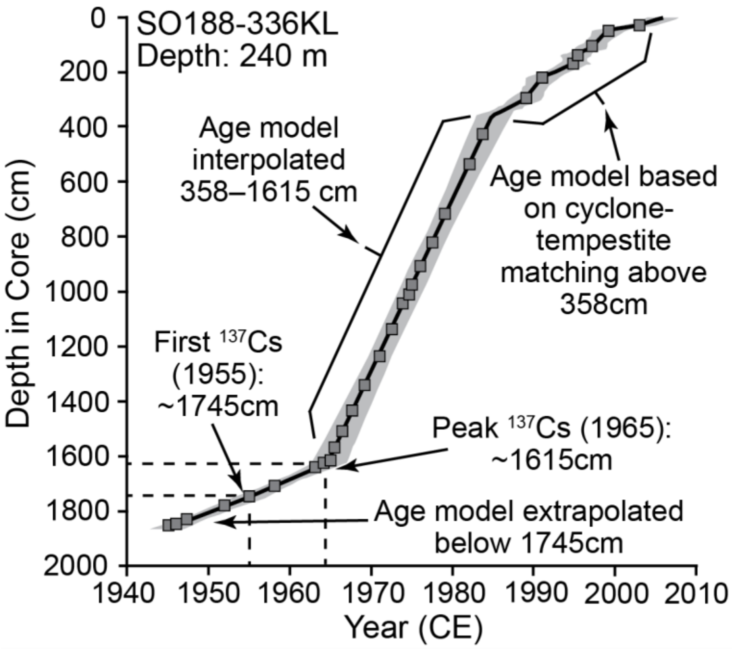

Figure S3. Core age profile determined from a combination of correlation of fining-upward tempestite beds in upper 358 cm (1985[8], as per methods described by Kudrass et al.[1]) and known Bay of Bengal cyclone impacts; interpolation between 358 cm and 1,615 cm (peak ^{137}Cs, 1965 CE[7]); interpolation between peak ^{137}Cs and onset of down-core ^{137}Cs at 1,745 cm (1955); and extrapolation from 1,745 cm to the core bottom (1,860 cm). Age model calculated using the Bayesian age-depth modeling software package Clam 2.1[34], with standard errors of ±1 year for each control date (error window shown in gray shading). Squares denote samples used in analysis.

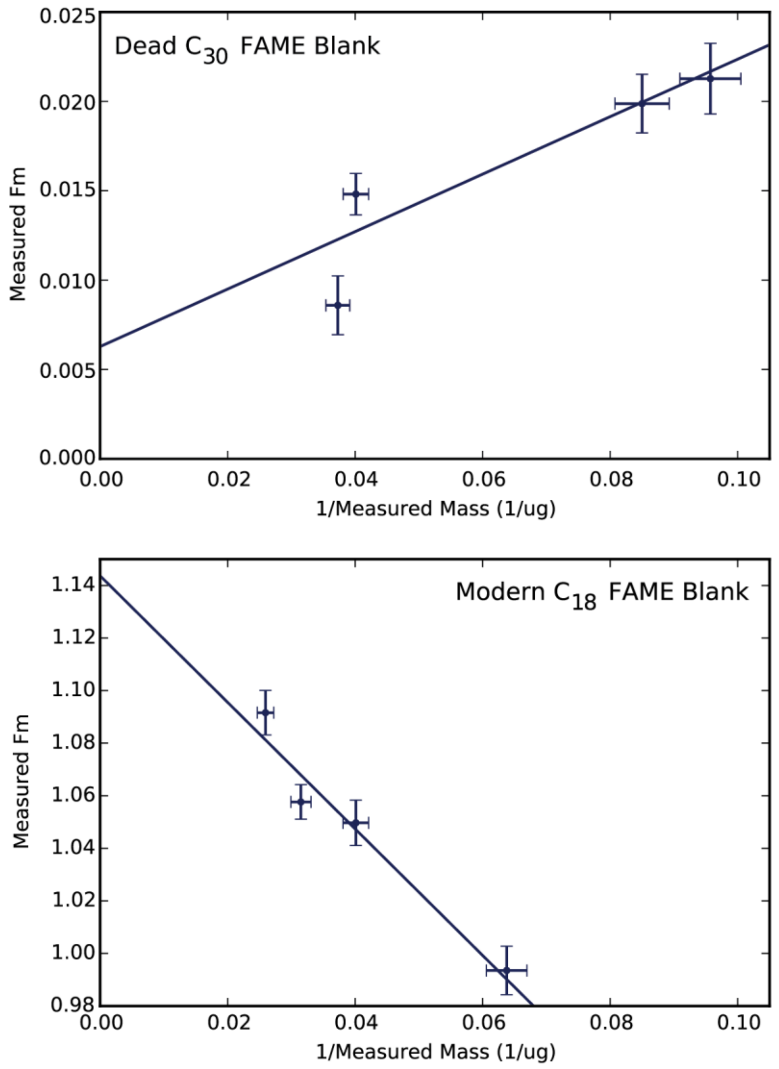

Figure S4. Graphical blank calculation where the top panel shows measured Fm for blanks spiked with radiocarbon dead *n*-C_{30} FAME vs. 1/measured C mass. Similarly, the bottom panel shows measured Fm vs. inverse measured C mass for blanks spiked with modern *n*-C_{18} FAME. The regression lines were calculated using an uncertainty-weighted Model II regression.

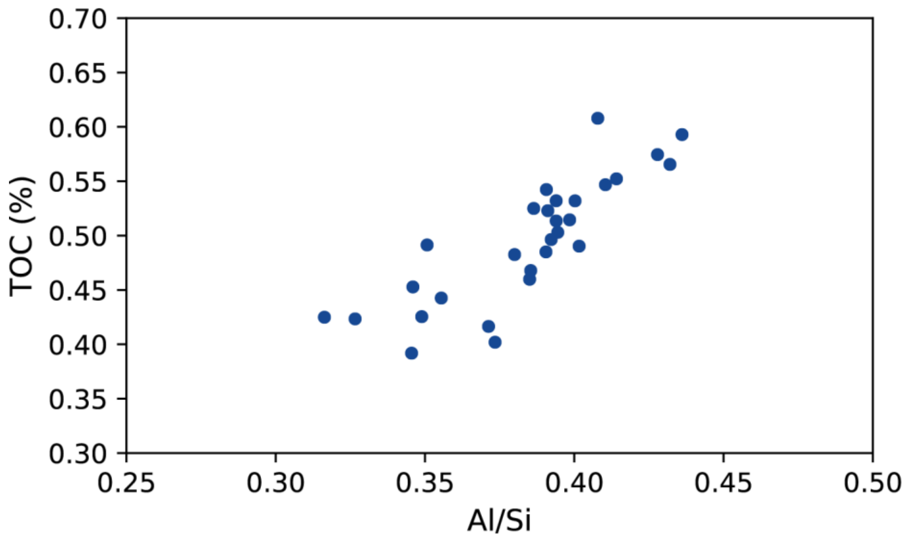

Figure S5. Crossplot of Al/Si vs. TOC for all SO188-336KL samples used in this study.

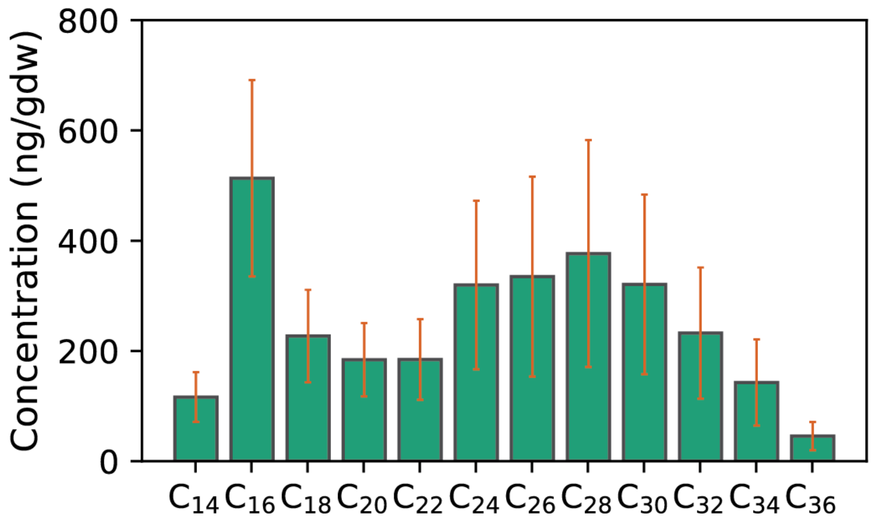

Figure S6. Average concentration of fatty acid methyl esters (FAMEs) showing bimodal distribution and prominence of long chain fatty acids. The average concentration for each fatty acid chain length was calculated from all sediment horizons from the core (see Table S2). FAME concentrations are reported relative to gram dry weight of sediment (gdw), and the error bars represent 1 standard deviation.

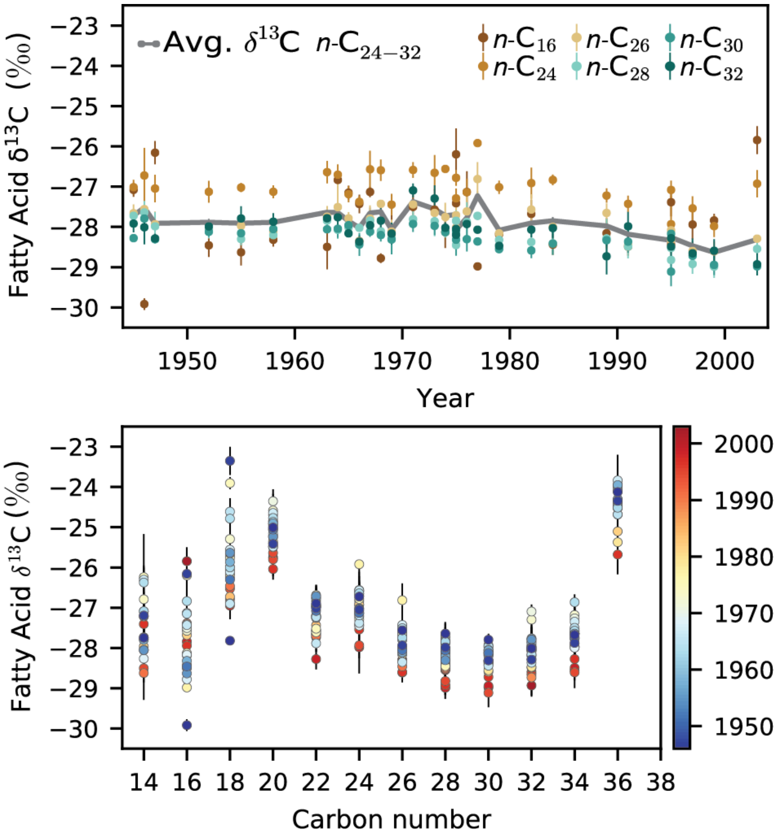

Figure S7. Stable carbon isotopic composition of *n*-C_{16}, *n*-C_{24}, *n*-C_{26}, *n*-C_{28}, *n*-C_{30}, and *n*-C_{32} fatty acids and concentration-weighted average of *n*-$C_{24\text{-}32}$ fatty acid δ^{13}C values plotted as a function of sample year (top). Even-numbered fatty acid δ^{13}C values are plotted as a function of carbon number and colored according to sample year (bottom). The error bars represent 1σ error.

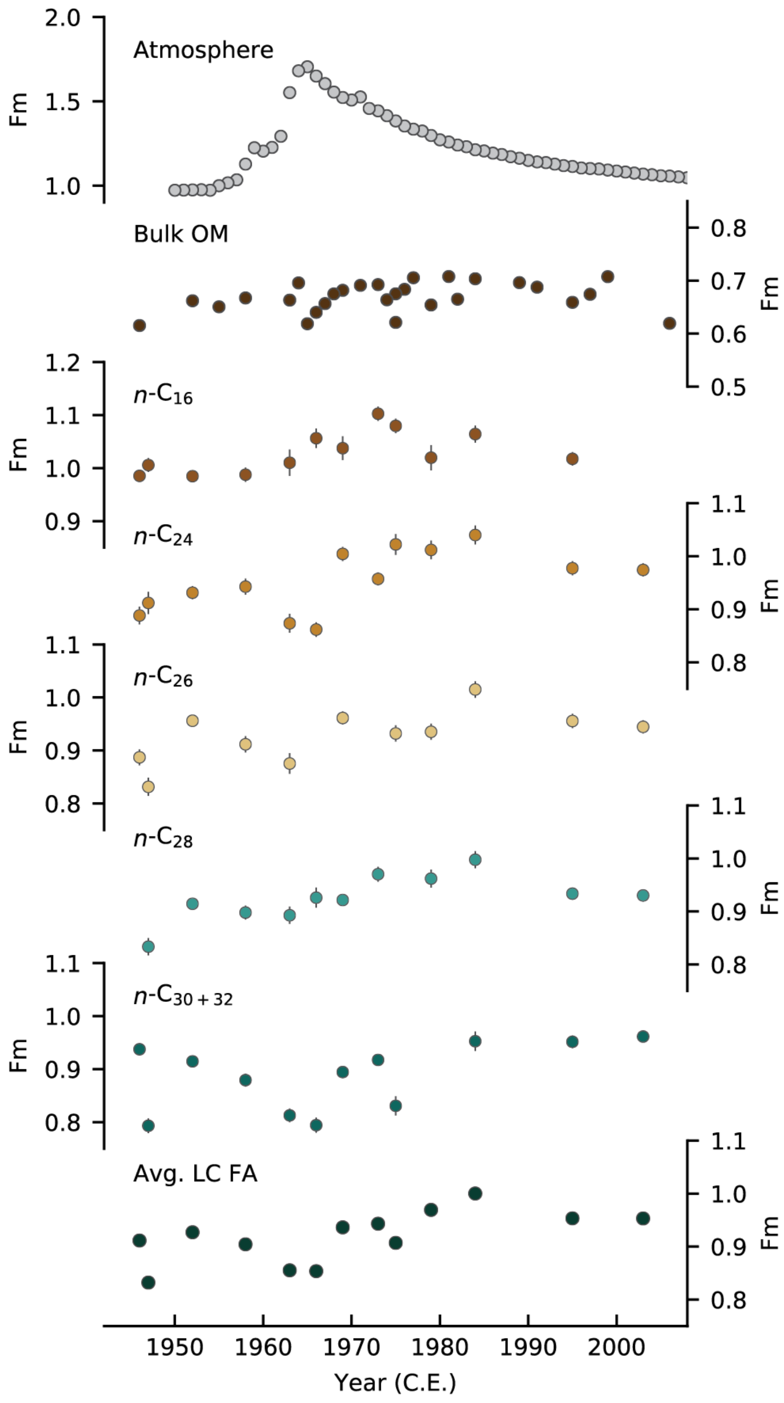

Figure S8. Radiocarbon composition of bulk OM and fatty acids reported in Fm compared to atmospheric radiocarbon composition in the northern hemisphere zone 3[7]. The bottom panel shows the concentration-weighted average long-chain fatty acid (Avg. LC FA) Fm. The error bars represent 1σ error.

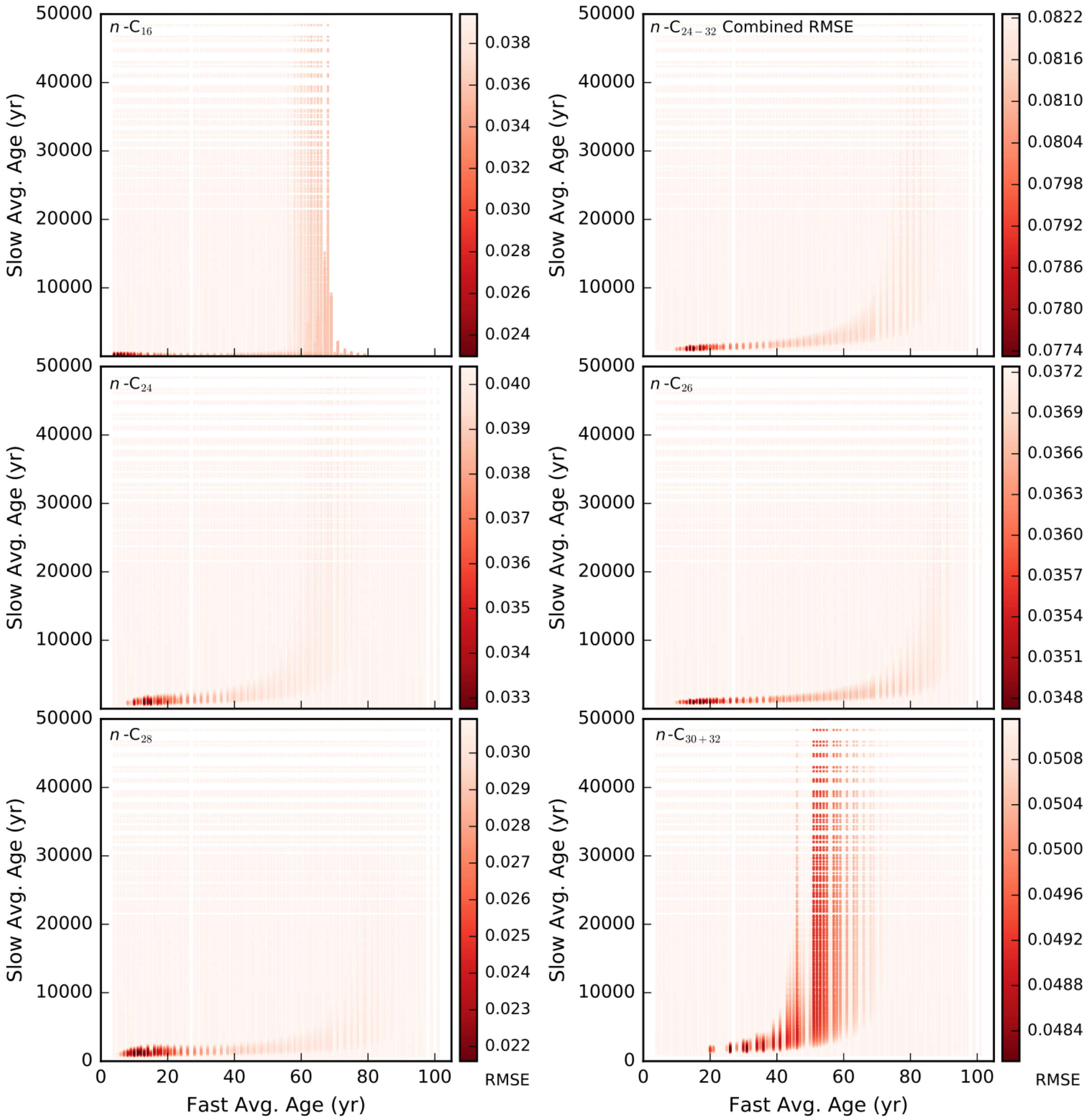

Figure S9. RMSE heat maps for *n*-C_{16}, *n*-C_{24}, *n*-C_{26}, *n*-C_{28}, *n*-C_{30+32}, and combined *n*-$C_{24\text{-}32}$ RMSE where the x-axis and y-axis scales are set to show the full solution space that was considered for the isotope mixing simulations. The color bar is scaled to bracket the top 10% RMSE values, so simulations outside of the top 10% are colored the same pale red.

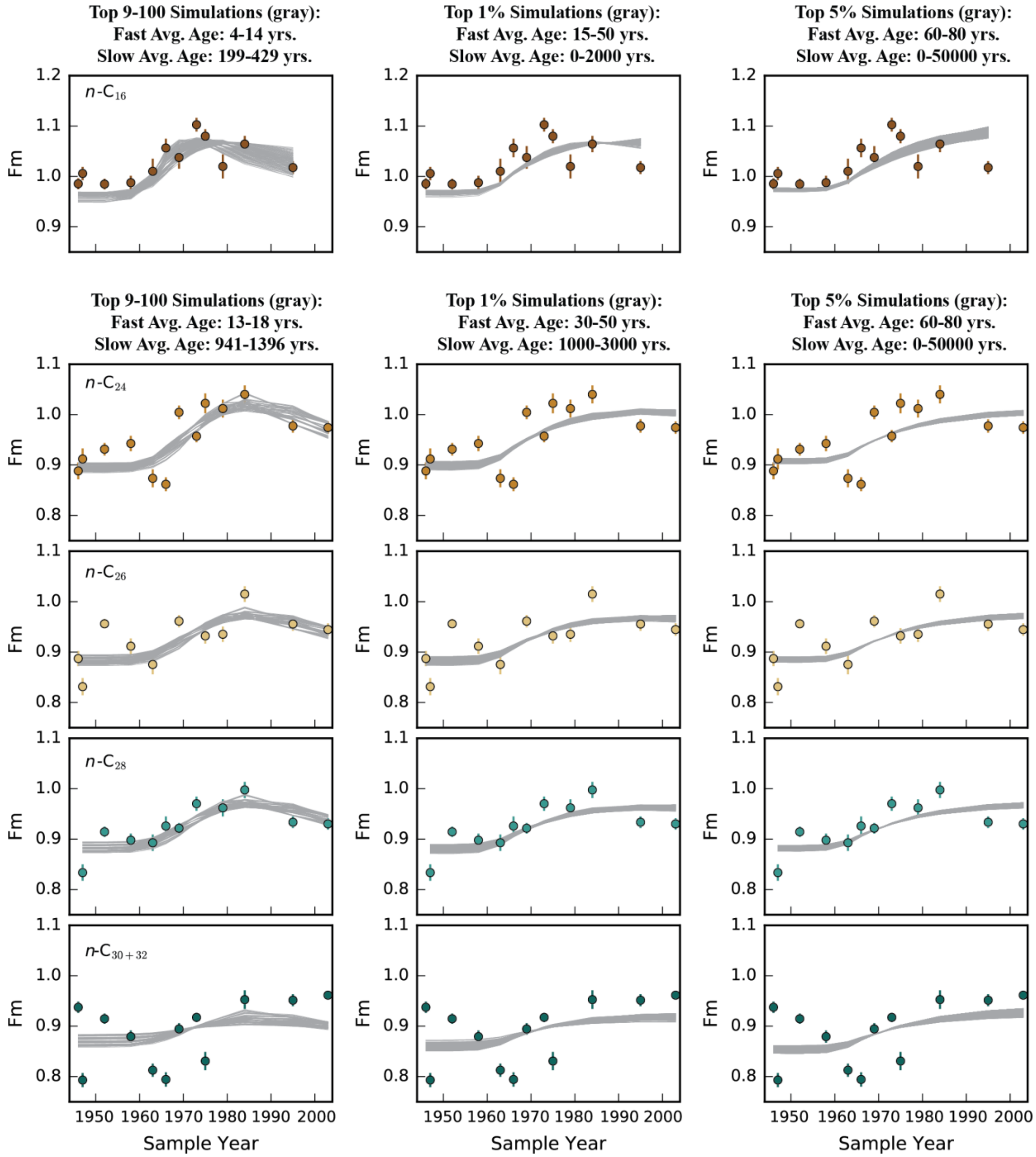

Figure S10. Less optimally fitting age distributions. Synthetic Fm time series (gray) are compared to measured fatty acid Fm data. Solutions that had the top 9-100 best fits were plotted in the first column. The second and third columns show solutions that are within the top 1% and 5% best fits, respectively, *and* are also within the specified average age ranges for that given column. The second and third columns show that he top 1-5% best fitting solutions that have significantly different fast- and slow-cycling average ages from the top 100 best fitting solutions do not achieve the full magnitude and recovery of the isotopic excursion.

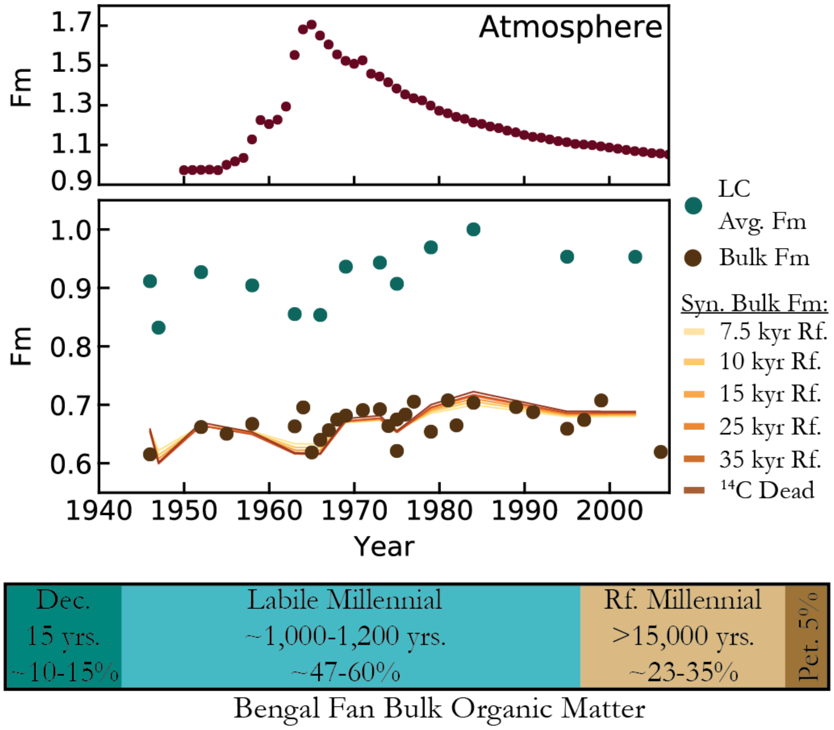

Figure S11. Bulk organic matter age structure. The concentration weighted average long-chain (LC) fatty acid Fm (green) is plotted with the measured bulk OM Fm (brown) and the atmospheric bomb spike for the northern hemisphere zone 3[7]. Synthetic bulk Fm values are plotted for different refractory millennial ages. The schematic illustrates fractional contributions of decadal (Dec.), labile millennial, refractory millennial (Rf.), and petrogenic (Pet.) carbon to the bulk OM.

Table S1. Bulk Geochemistry including bulk organic carbon (OC) radiocarbon Fm. Bulk OC Fm values are marked in bold if they represent average values of duplicates with propagated error. The average precision (2σ) of TOC replicate measurements is 0.02%. Average uncertainties (2σ) of major/trace elemental compositions and of $^{87}Sr/^{86}Sr$ and εNd isotopic compositions are better than 2% (relative), $2x10^{-5}$, and 0.5 ε units, respectively.

Sample ID	Year	Median Grain Size (mm)	TOC (%)	Bulk OM Fm	Bulk OM Fm (1σ)	Al/Si	$^{143}Nd/^{144}Nd$	2 s.d.	εNd	2 s.d.	$^{87}Sr/^{86}Sr$	2 s.d
0-1 cm (core top)	2006		0.39	0.6194	0.0022							
25-35 cm	2003	20.49	0.49			0.35	0.51191	7.E-06	-14.1	0.26	0.74539	7.E-06
45-55 cm	1999	23.62	0.54	0.7075	0.0099	0.39						
111-112 cm	1997		0.42	0.6742	0.0084	0.32						
135-145 cm	1995	22.99	0.57			0.43	0.51189	4.E-06	-14.7	0.15	0.74367	2.E-05
164-174 cm	1995	22.69	0.52	**0.6591**	**0.0060**	0.39						
215-225 cm	1991	19.62	0.59	**0.6877**	**0.0062**	0.44						
292-302 cm	1989	18.12	0.53	0.6962	0.0094	0.39	0.51190	6.E-06	-14.4	0.23	0.74665	1.E-05
420-430 cm	1984	21.31	0.55	**0.7036**	**0.0063**	0.41						
530-540 cm	1982	20.45	0.52	0.6650	0.0084	0.39	0.51191	5.E-06	-14.1	0.19	0.74299	9.E-06
602-612 cm	1981	13.96	0.55	**0.7077**	**0.0058**	0.41						
705-715 cm	1979	17.00	0.42	0.6541	0.0080	0.33						
815-825 cm	1977	21.57	0.50	**0.7055**	**0.0065**	0.39						
905-915 cm	1976	16.80	0.51	0.6835	0.0089	0.40	0.51191	5.E-06	-14.2	0.20	0.74604	2.E-05
970-980 cm	1975	19.36	0.53	0.6751	0.0092	0.40						
1014-1020 cm	1975		0.39	**0.6212**	**0.0056**	0.35						
1035-1045 cm	1974	19.55	0.46	0.6639	0.0095	0.39						
1130-1140 cm	1973	17.55	0.57	0.6924	0.0086	0.43	0.51188	4.E-06	-14.9	0.16	0.74844	2.E-05
1239-1249 cm	1971	11.43	0.49	**0.6910**	**0.0060**	0.40						
1330-1340 cm	1969	17.62	0.50	0.6817	0.0083	0.39						
1425-1435 cm	1968	15.56	0.49	**0.6750**	**0.0061**	0.39						
1505-1515 cm	1967	16.56	0.47	0.6566	0.0082	0.39						
1570-1580 cm	1966		0.48	0.6402	0.0037	0.38	0.51190	6.E-06	-14.5	0.22	0.74321	1.E-05
1614-1620 cm	1965	25.08	0.42	**0.6187**	**0.0057**	0.37						
1620-1627 cm	1964	22.78	0.40	0.6957	0.0086	0.37						
1635-1645 cm	1963	19.36	0.44	0.6632	0.0081	0.36						
1700-1710 cm	1958	16.20	0.51	**0.6673**	**0.0058**	0.39						
1741-1746 cm	1955	16.01	0.45	0.6506	0.0077	0.35						
1770-1780 cm	1952	15.95	0.61	0.6620	0.0101	0.41						
1820-1828 cm	1947											
1832-1842 cm	1946	19.46	0.43	0.6153	0.0084	0.35						

Table S2. FAME concentrations normalized to grams dry weight (gdw) of sediment.

**Sample ID**	**Year**	**C_{14} FA (ng/gdw)**	**C_{16} FA (ng/gdw)**	**C_{18} FA (ng/gdw)**	**C_{20} FA (ng/gdw)**	**C_{22} FA (ng/gdw)**	**C_{24} FA (ng/gdw)**	**C_{26} FA (ng/gdw)**	**C_{28} FA (ng/gdw)**	**C_{30} FA (ng/gdw)**	**C_{32} FA (ng/gdw)**	**C_{34} FA (ng/gdw)**	**C_{36} FA (ng/gdw)**	**C_{14-36} Sum (ng/gdw)**
25-35 cm	2003	252	963	417	229	237	459	481	553	490	363	225	69	4737
45-55 cm	1999	204	876	446	337	362	715	812	870	617	382	222	70	5912
111-112 cm	1997	96	713	355	239	210	318	305	347	295	227	153	51	3307
135-145 cm	1995		534	314	303	349	706	854	1020	847	618	393	128	6067
164-174 cm	1995	173	717	263	222	244	436	433	508	448	351	229	74	4097
215-225 cm	1991	138	596	254	233	245	395	402	472	420	332	217	74	3778
292-302 cm	1989	158	619	239	193	202	336	330	356	297	193	105	33	3060
420-430 cm	1984	117	530	214	174	182	335	310	295	213	151	104	35	2658
530-540 cm	1982	180	649	262	180	176	274	261	296	249	177	101	38	2842
705-715 cm	1979	76	310	146	144	141	234	239	252	208	150	95	33	2029
815-825 cm	1977	80	630	258	124	136	317	327	314	239	151	80	20	2677
905-915 cm	1976	117	501	223	189	196	329	349	414	375	283	173	56	3204
970-980 cm	1975	113	482	199	180	184	296	301	341	315	231	133	42	2817
1014-1020 cm	1975	108	453	204	127	130	199	196	219	208	165	99	33	2141
1035-1045 cm	1974	101	386	153	149	154	265	278	316	275	190	107	32	2405
1130-1140 cm	1973	110	463	191	204	215	368	395	448	390	281	160	51	3275
1239-1249 cm	1971	69	302	153	131	134	228	233	241	195	135	81	23	1924
1330-1340 cm	1969	76	322	145	172	163	273	295	336	292	209	125	40	2446
1425-1435 cm	1968	97	389	156	137	136	231	241	265	236	173	99	30	2190
1505-1515 cm	1967	73	293	140	131	130	217	219	252	229	163	89	28	1964
1570-1580 cm	1966	125	510	213	149	139	208	179	159	128	94	69	14	1987
1614-1620 cm	1965	87	371	182	146	127	197	209	252	247	199	128	44	2190
1620-1627 cm	1964	91	351	149	118	119	188	196	237	214	144	75	23	1904
1635-1645 cm	1963	57	252	105	91	85	138	149	191	180	120	65	20	1453
1700-1710 cm	1958	95	418	173	176	168	261	267	298	240	161	93	27	2377
1741-1746 cm	1955	96	433	202	172	167	273	300	381	347	279	184	59	2894
1770-1780 cm	1952	164	787	362	385	373	688	750	814	677	500	329	101	5930
1820-1828 cm	1947	119	462	226	165	118	182	195	243	217	161	116	42	2245
1832-1842 cm	1946	86	570	241	136	132	203	203	233	213	158	91	27	2294
**Average**		116	513	227	184	185	320	335	377	321	232	143	45	2993
**Standard Deviation**		45	178	84	66	73	153	181	206	163	119	78	26	1250

Table S3. Stable carbon isotopic data for fatty acids in ‰ listed with 1σ error.

Sample ID	Year	Corrected C_{14} FA $\delta^{13}C$	Corrected C_{16} FA $\delta^{13}C$	Corrected C_{18} FA $\delta^{13}C$	Corrected C_{20} FA $\delta^{13}C$	Corrected C_{22} FA $\delta^{13}C$	Corrected C_{24} FA $\delta^{13}C$	Corrected C_{26} FA $\delta^{13}C$	Corrected C_{28} FA $\delta^{13}C$	Corrected C_{30} FA $\delta^{13}C$	Corrected C_{32} FA $\delta^{13}C$	Corrected C_{34} FA $\delta^{13}C$	Weighted Avg. C_{24-32} FA $\delta^{13}C$
25-35 cm	2003		-25.8 +/- 0.3	-26.0 +/- 0.3	-25.8 +/- 0.5	-27.7 +/- 0.1	-26.9 +/- 0.3	-28.3 +/- 0.1	-28.5 +/- 0.3	-29.0 +/- 0.2	-28.9 +/- 0.3	-28.6 +/- 0.2	-28.3 +/- 0.1
45-55 cm	1999		-27.8 +/- 0.2	-26.9 +/- 0.3	-25.5 +/- 0.4	-28.3 +/- 0.3	-28.0 +/- 0.3	-28.6 +/- 0.2	-29.0 +/- 0.3	-28.9 +/- 0.3	-28.6 +/- 0.3	-28.5 +/- 0.2	-28.6 +/- 0.1
111-112 cm	1997	-27.4 +/- 0.4	-27.9 +/- 0.4	-26.3 +/- 0.4	-26.0 +/- 0.3	-27.6 +/- 0.1	-27.5 +/- 0.3	-28.6 +/- 0.1	-28.9 +/- 0.2	-28.7 +/- 0.3	-28.7 +/- 0.4	-28.3 +/- 0.3	-28.5 +/- 0.1
135-145 cm	1995		-28.4 +/- 0.1	-26.8 +/- 0.2	-25.8 +/- 0.3	-27.7 +/- 0.0	-27.9 +/- 0.7	-28.3 +/- 0.1	-28.5 +/- 0.1	-28.2 +/- 0.2	-28.3 +/- 0.2	-28.6 +/- 0.4	-28.3 +/- 0.1
164-174 cm	1995	-28.5 +/- 0.8	-27.4 +/- 0.3	-26.5 +/- 0.3	-25.6 +/- 0.3	-27.6 +/- 0.4	-27.1 +/- 0.2	-28.1 +/- 0.3	-28.8 +/- 0.3	-29.1 +/- 0.4	-28.5 +/- 0.3	-27.9 +/- 0.3	-28.3 +/- 0.1
215-225 cm	1991	-28.6 +/- 0.0	-28.5 +/- 0.2	-26.5 +/- 0.4	-25.5 +/- 0.2	-27.7 +/- 0.2	-27.4 +/- 0.2	-28.5 +/- 0.1	-28.5 +/- 0.3	-28.4 +/- 0.3	-28.0 +/- 0.4	-27.8 +/- 0.5	-28.2 +/- 0.1
292-302 cm	1989	-27.2 +/- 0.3	-28.2 +/- 0.4	-25.7 +/- 0.3	-25.2 +/- 0.6	-27.2 +/- 0.8	-27.2 +/- 0.2	-27.7 +/- 0.2	-28.3 +/- 0.5	-28.3 +/- 0.1	-28.7 +/- 0.4	-28.0 +/- 0.5	-28.0 +/- 0.1
420-430 cm	1984		-28.4 +/- 0.3	-26.7 +/- 0.1	-25.1 +/- 0.3	-27.4 +/- 0.2	-26.8 +/- 0.1	-28.0 +/- 0.1	-28.4 +/- 0.3	-28.4 +/- 0.3	-28.0 +/- 0.2	-27.7 +/- 0.3	-27.8 +/- 0.1
530-540 cm	1982	-28.0 +/- 0.6	-27.7 +/- 0.3	-26.2 +/- 0.4	-25.4 +/- 0.7	-27.6 +/- 0.5	-26.9 +/- 0.4	-27.6 +/- 0.4	-28.4 +/- 0.3	-28.6 +/- 0.2	-28.1 +/- 0.2	-28.0 +/- 0.7	-27.9 +/- 0.1
705-715 cm	1979	-28.0 +/- 0.1	-28.2 +/- 0.1	-26.2 +/- 0.3	-25.4 +/- 0.1	-27.2 +/- 0.3	-27.0 +/- 0.2	-28.2 +/- 0.0	-28.3 +/- 0.2	-28.6 +/- 0.1	-28.5 +/- 0.1	-28.0 +/- 0.1	-28.1 +/- 0.1
815-825 cm	1977		-29.0 +/- 0.1	-26.9 +/- 0.1	-25.0 +/- 0.2	-26.7 +/- 0.2	-25.9 +/- 0.0	-26.8 +/- 0.4	-27.7 +/- 0.1	-28.4 +/- 0.1	-28.1 +/- 0.0	-27.6 +/- 0.3	-27.2 +/- 0.1
905-915 cm	1976	-27.7 +/- 0.4	-27.1 +/- 0.5	-25.6 +/- 0.2	-24.9 +/- 0.4	-26.8 +/- 0.4	-27.1 +/- 0.3	-27.6 +/- 0.4	-27.9 +/- 0.5	-28.3 +/- 0.3	-27.9 +/- 0.1	-27.6 +/- 0.2	-27.8 +/- 0.2
970-980 cm	1975	-28.0 +/- 0.3	-27.4 +/- 0.4	-25.8 +/- 0.5	-25.0 +/- 0.2	-27.6 +/- 0.2	-27.3 +/- 0.2	-28.0 +/- 0.5	-28.5 +/- 0.0	-28.3 +/- 0.4	-28.2 +/- 0.3	-27.6 +/- 0.2	-28.1 +/- 0.1
1014-1020 cm	1975	-26.2 +/- 0.2	-26.2 +/- 0.6	-23.9 +/- 0.1	-24.9 +/- 0.5	-27.5 +/- 0.1	-26.8 +/- 0.2	-27.7 +/- 0.4	-27.8 +/- 0.3	-28.1 +/- 0.3	-28.1 +/- 0.3	-27.6 +/- 0.6	-27.7 +/- 0.1
1035-1045 cm	1974	-26.8 +/- 0.5	-28.1 +/- 0.4	-25.7 +/- 0.3	-24.8 +/- 0.3	-27.0 +/- 0.2	-26.6 +/- 0.1	-27.8 +/- 0.4	-28.1 +/- 0.1	-28.1 +/- 0.0	-28.0 +/- 0.1	-27.6 +/- 0.2	-27.7 +/- 0.1
1130-1140 cm	1973	-27.8 +/- 0.9	-27.6 +/- 0.5	-25.3 +/- 0.5	-24.6 +/- 0.3	-27.3 +/- 0.2	-26.7 +/- 0.4	-27.7 +/- 0.3	-27.9 +/- 0.4	-28.0 +/- 0.2	-27.3 +/- 0.3	-27.2 +/- 0.4	-27.6 +/- 0.2
1239-1249 cm	1971		-27.5 +/- 0.3	-25.6 +/- 0.3	-24.4 +/- 0.2	-27.0 +/- 0.1	-26.6 +/- 0.2	-27.4 +/- 0.1	-27.8 +/- 0.2	-27.9 +/- 0.2	-27.1 +/- 0.2	-27.3 +/- 0.2	-27.4 +/- 0.1
1330-1340 cm	1969	-28.3 +/- 0.6	-28.2 +/- 0.5	-26.1 +/- 0.4	-25.2 +/- 0.2	-27.2 +/- 0.4	-27.4 +/- 0.3	-28.1 +/- 0.3	-28.3 +/- 0.2	-28.3 +/- 0.4	-28.2 +/- 0.3	-28.0 +/- 0.3	-28.1 +/- 0.1
1425-1435 cm	1968	-27.1 +/- 0.2	-28.8 +/- 0.1	-25.6 +/- 0.6	-24.7 +/- 0.2	-26.8 +/- 0.2	-26.6 +/- 0.3	-27.4 +/- 0.1	-28.1 +/- 0.1	-28.2 +/- 0.2	-27.8 +/- 0.4	-27.5 +/- 0.4	-27.6 +/- 0.1
1505-1515 cm	1967	-26.3 +/- 1.1	-27.1 +/- 0.1	-26.1 +/- 0.2	-24.6 +/- 0.6	-26.9 +/- 0.2	-26.6 +/- 0.5	-27.8 +/- 0.3	-27.8 +/- 0.2	-28.1 +/- 0.1	-28.0 +/- 0.1	-27.3 +/- 0.5	-27.7 +/- 0.1
1570-1580 cm	1966		-27.4 +/- 0.2	-26.9 +/- 0.3	-25.5 +/- 0.2	-27.9 +/- 0.1	-27.4 +/- 0.1	-28.3 +/- 0.1	-28.0 +/- 0.1	-28.4 +/- 0.3	-28.4 +/- 0.1	-27.8 +/- 0.1	-28.0 +/- 0.1
1614-1620 cm	1965	-27.2 +/- 0.6	-27.2 +/- 0.2	-24.6 +/- 0.3	-25.2 +/- 0.5	-27.2 +/- 0.2	-27.2 +/- 0.2	-27.8 +/- 0.2	-28.0 +/- 0.1	-28.0 +/- 0.2	-28.2 +/- 0.1	-27.5 +/- 0.2	-27.9 +/- 0.1
1620-1627 cm	1964	-26.4 +/- 0.4	-26.8 +/- 0.4	-24.8 +/- 0.1	-24.8 +/- 0.5	-27.0 +/- 0.3	-26.7 +/- 0.3	-27.5 +/- 0.8	-28.1 +/- 0.2	-28.0 +/- 0.2	-27.8 +/- 0.3	-26.9 +/- 0.2	-27.6 +/- 0.2
1635-1645 cm	1963	-27.1 +/- 0.1	-28.5 +/- 0.6	-26.0 +/- 0.4	-24.9 +/- 0.6	-26.9 +/- 0.3	-26.6 +/- 0.3	-27.7 +/- 0.3	-27.8 +/- 0.3	-28.1 +/- 0.1	-27.8 +/- 0.2	-27.6 +/- 0.6	-27.6 +/- 0.1
1700-1710 cm	1958	-27.8 +/- 0.5	-28.3 +/- 0.2	-25.9 +/- 0.3	-24.9 +/- 0.4	-26.8 +/- 0.2	-27.1 +/- 0.2	-28.1 +/- 0.1	-28.2 +/- 0.1	-28.1 +/- 0.2	-27.9 +/- 0.2	-27.6 +/- 0.4	-27.9 +/- 0.1
1741-1746 cm	1955	-28.1 +/- 0.4	-28.6 +/- 0.3	-25.6 +/- 0.3	-25.2 +/- 0.1	-26.7 +/- 0.3	-27.0 +/- 0.1	-28.0 +/- 0.2	-28.3 +/- 0.2	-28.2 +/- 0.2	-27.8 +/- 0.3	-27.6 +/- 0.2	-27.9 +/- 0.1
1770-1780 cm	1952		-28.5 +/- 0.3	-26.3 +/- 0.2	-25.1 +/- 0.2	-27.1 +/- 0.1	-27.1 +/- 0.3	-28.1 +/- 0.1	-28.1 +/- 0.2	-28.1 +/- 0.1	-28.0 +/- 0.1	-27.7 +/- 0.3	-27.9 +/- 0.1
1820-1828 cm	1947	-27.2 +/- 0.4	-26.2 +/- 0.3	-23.4 +/- 0.3	-25.4 +/- 0.3	-27.0 +/- 0.4	-27.0 +/- 0.3	-27.9 +/- 0.3	-28.0 +/- 0.4	-28.3 +/- 0.1	-28.3 +/- 0.1	-27.9 +/- 0.5	-27.9 +/- 0.1
1832-1842 cm	1946	-27.7 +/- 0.4	-29.9 +/- 0.1	-27.8 +/- 0.1	-25.0 +/- 0.3	-26.9 +/- 0.1	-26.7 +/- 0.7	-27.6 +/- 0.2	-27.6 +/- 0.3	-27.8 +/- 0.1	-28.0 +/- 0.4	-27.7 +/- 0.2	-27.5 +/- 0.2

Table S4. Raw fatty acid radiocarbon fraction modern (Fm) listed with 1σ error. Anomalously contaminated samples are italicized in gray and were not included in isotope modeling.

Sample	Year	C_{16} Raw Fm	C_{16} Meas Mass µg C	C_{24} Raw Fm	C_{24} Meas Mass µg C	C_{26} Raw Fm	C_{26} Meas Mass µg C	C_{28} Raw Fm	C_{28} Meas Mass µg C	C_{30} Raw Fm	C_{30} Meas Mass µg C	C_{30+32} Raw Fm	C_{30+32} Meas Mass µg C	C_{32} Raw Fm	C_{32} Meas Mass µg C
25-35 cm	2003			0.8943 +/- 0.0078	46.3	0.8714 +/- 0.0086	48.3	0.8583 +/- 0.0068	45.7	0.8714 +/- 0.0074	49.3			0.9126 +/- 0.0090	44.3
164-174 cm	1995	0.9075 +/- 0.0058	38.9	0.8856 +/- 0.0060	36.2	0.8665 +/- 0.0063	34.8	0.8562 +/- 0.0057	40.5	0.8640 +/- 0.0055	36.5			0.8244 +/- 0.0083	17.0
292-302 cm	1989	*0.8157 +/- 0.0059*	*58.1*	*0.7955 +/- 0.0058*	*51.6*	*0.7354 +/- 0.0056*	*47.9*	*0.7165 +/- 0.0056*	*53.4*	*0.6821 +/- 0.0053*	*38.6*			*0.6064 +/- 0.0052*	*27.4*
420-430 cm	1984	0.9421 +/- 0.0084	34.4	0.9285 +/- 0.0081	29.2	0.9186 +/- 0.0082	33.9	0.8985 +/- 0.0072	30.4			0.8480 +/- 0.0077	25.2		
705-715 cm	1979	0.8629 +/- 0.0070	20.2	0.8978 +/- 0.0065	26.9	0.8379 +/- 0.0060	29.1	0.8594 +/- 0.0069	27.2						
905-915 cm	1976	*0.7607 +/- 0.0054*	*39.8*	*0.7111 +/- 0.0058*	*36.5*	*0.7219 +/- 0.0056*	*47.7*	*0.7469 +/- 0.0055*	*60.5*						
970-980 cm	1975	0.9757 +/- 0.0096	51.4	0.9019 +/- 0.0079	25.2	0.8367 +/- 0.0070	29.9	*0.5719 +/- 0.0066*	*57.1*			0.7317 +/- 0.0071	22.1		
1130-1140 cm	1973	0.9845 +/- 0.0064	40.0	0.8718 +/- 0.0063	39.6			0.8787 +/- 0.0059	32.8			0.8564 +/- 0.0060	56.8		
1330-1340 cm	1969	0.8859 +/- 0.0073	22.0	0.9111 +/- 0.0064	36.9	0.8786 +/- 0.0064	39.9	0.8497 +/- 0.0058	44.9			0.8210 +/- 0.0056	38.9		
1505-1515 cm	1967	*0.7270 +/- 0.0054*	*34.3*			*0.7002 +/- 0.0057*	*35.4*	*0.6427 +/- 0.0050*	*43.9*			*0.6029 +/- 0.0049*	*59.6*		
1570-1580 cm	1966	0.9261 +/- 0.0090	29.8	0.7798 +/- 0.0082	35.0			0.8201 +/- 0.0086	24.5	0.6674 +/- 0.0077	24.1			0.7323 +/- 0.0100	16.7
1635-1645 cm	1963	0.8514 +/- 0.0081	19.5	0.7671 +/- 0.0068	23.6	0.7667 +/- 0.0083	22.3	0.7961 +/- 0.0066	26.4			0.7345 +/- 0.0055	29.9		
1700-1710 cm	1958	0.8757 +/- 0.0061	35.1	0.8434 +/- 0.0062	29.8	0.8166 +/- 0.0069	29.0	0.8133 +/- 0.0059	32.7			0.8025 +/- 0.0055	35.1		
1770-1780 cm	1952	0.9050 +/- 0.0086	85.9	0.8616 +/- 0.0096	55.8	0.8991 +/- 0.0076	86.4	0.8614 +/- 0.0087	83.0	0.8449 +/- 0.0091	58.1			0.8299 +/- 0.0077	21.8
1820-1828 cm	1947	0.9039 +/- 0.0083	45.1	0.7917 +/- 0.0079	21.2	0.7325 +/- 0.0068	23.6	0.7389 +/- 0.0065	24.5			0.7217 +/- 0.0087	33.1		
1832-1842 cm	1946	0.9085 +/- 0.0094	99.4	0.7991 +/- 0.0107	32.0	0.7983 +/- 0.0079	30.8			0.7861 +/- 0.0068	31.2			0.9111 +/- 0.0121	47.2

Table S5. Corrected fatty acid radiocarbon fraction modern (Fm) listed with 1σ error. The raw fraction modern was corrected for blank contribution and methylation. The italicized data are affected by contamination and therefore excluded from further data analysis.

Sample	Year	C_{16} Corrected Fm	C_{24} Corrected Fm	C_{26} Corrected Fm	C_{28} Corrected Fm	C_{30} Corrected Fm	C_{30+32} Corrected Fm	C_{32} Corrected Fm
25-35 cm	2003		0.9743 +/- 0.0122	0.9446 +/- 0.0123	0.9302 +/- 0.0112	0.9391 +/- 0.0111	0.9615 +/- 0.0087	0.9865 +/- 0.0134
164-174 cm	1995	1.0175 +/- 0.0127	0.9775 +/- 0.0131	0.9556 +/- 0.0134	0.9336 +/- 0.0113	0.9454 +/- 0.0123	0.9516 +/- 0.0119	0.9658 +/- 0.0273
292-302 cm	1989	*0.8978 +/- 0.0090*	*0.8623 +/- 0.0093*	*0.7969 +/- 0.0091*	*0.7709 +/- 0.0084*	*0.7430 +/- 0.0098*	*0.7146 +/- 0.0077*	*0.6736 +/- 0.0118*
420-430 cm	1984	1.0642 +/- 0.0160	1.0401 +/- 0.0177	1.0151 +/- 0.0155	0.9976 +/- 0.0160		0.9527 +/- 0.0184	
705-715 cm	1979	1.0197 +/- 0.0237	1.0120 +/- 0.0176	0.9353 +/- 0.0152	0.9620 +/- 0.0169			
905-915 cm	1976	*0.8512 +/- 0.0107*	*0.7836 +/- 0.0109*	*0.7824 +/- 0.0090*	*0.8001 +/- 0.0080*			
970-980 cm	1975	1.0797 +/- 0.0137	1.0224 +/- 0.0196	0.9321 +/- 0.0153	*0.6131 +/- 0.0083*		0.8308 +/- 0.0181	
1130-1140 cm	1973	1.1026 +/- 0.0135	0.9573 +/- 0.0122		0.9703 +/- 0.0140		0.9174 +/- 0.0091	
1330-1340 cm	1969	1.0376 +/- 0.0223	1.0045 +/- 0.0133	0.9613 +/- 0.0122	0.9216 +/- 0.0105		0.8945 +/- 0.0113	
1505-1515 cm	1967	*0.8202 +/- 0.0116*		*0.7705 +/- 0.0110*	*0.6969 +/- 0.0084*		*0.6438 +/- 0.0068*	
1570-1580 cm	1966	1.0564 +/- 0.0182	0.8618 +/- 0.0137		0.9260 +/- 0.0189	0.7516 +/- 0.0159	0.7943 +/- 0.0142	0.8588 +/- 0.0259
1635-1645 cm	1963	1.0102 +/- 0.0248	0.8736 +/- 0.0177	0.8754 +/- 0.0194	0.8928 +/- 0.0162		0.8128 +/- 0.0129	
1700-1710 cm	1958	0.9876 +/- 0.0136	0.9427 +/- 0.0150	0.9117 +/- 0.0154	0.8979 +/- 0.0132		0.8793 +/- 0.0120	
1770-1780 cm	1952	0.9848 +/- 0.0105	0.9313 +/- 0.0124	0.9561 +/- 0.0092	0.9145 +/- 0.0102	0.9046 +/- 0.0116	0.9147 +/- 0.0102	0.9436 +/- 0.0207
1820-1828 cm	1947	1.0058 +/- 0.0131	0.9120 +/- 0.0208	0.8314 +/- 0.0170	0.8336 +/- 0.0163		0.7932 +/- 0.0138	
1832-1842 cm	1946	0.9854 +/- 0.0110	0.8883 +/- 0.0166	0.8871 +/- 0.0149		0.8685 +/- 0.0139	0.9375 +/- 0.0111	0.9819 +/- 0.0156

Table S6. Raw data for radiocarbon blanks. The raw fraction modern (Fm) is listed with 1σ error. The mass error was assumed to be 5% of the measured mass for error propagation.

Experiment	Injections	Spiked FAME	Predetermined Fm	Spiked FAME Mass μg	Measured Mass C μg	Measured Fm
1	110	*n*-C_{18}	1.1124	10	15.7	0.9935 +/- 0.0093
				25		*contaminated*
				40	31.8	1.0577 +/- 0.0066
		n-C_{30}	0	10	11.8	0.0199 +/- 0.0016
				25	26.8	0.0086 +/- 0.0016
				40		*contaminated*
2	80	*n*-C_{18}	1.1124	10		*lost*
				25	24.9	1.0497 +/- 0.0086
				40	38.6	1.0916 +/- 0.0085
		n-C_{30}	0	10	10.4	0.0213 +/- 0.0020
				25	24.9	0.0148 +/- 0.0012
				40		*contaminated*

Table S7. Radiocarbon blank regression parameters from graphical blank calculations.

Regression Parameter	*n*-C_{18} FAME	*n*-C_{30} FAME
**Slope (m)**	-2.4074	0.1608
**Intercept (b)**	1.1437	0.0063
**r^2**	0.9560	0.8232
**Slope standard deviation**	0.4808	0.0655
**Intercept standard deviation**	0.0186	0.0040

Table S8. Combined radiocarbon blank from the modern and dead blank components. The blank masses and fraction moderns (Fm) are listed with 1σ error.

	Blank Mass µg	Blank Mass Error µg	Blank Fm	Blank Fm Error
**Modern**	0.2	0.1	1.00	0.00
**Dead**	2.1	0.4	0.00	0.00
**Combined**	2.2	0.4	0.07	0.03

Table S9. Top 100 best fitting age distributions according to RMSE.

FA	σ_{Fast} *(yrs)*	σ_{Slow} *(yrs)*	μ_{Fast} *(yrs)*	μ_{Slow} *(yrs)*	*Fast Avg. Age (yrs)*	*Slow Avg. Age (yrs)*	f_{Slow}
C_{16}	5-17.5	250-500	0-9	0-400	4-14	199-429	0.75-0.86
C_{24}	5-12.5	500-1750	10-16	0-1200	10-17	710-1396	0.66-0.82
C_{26}	7.5-25	500-1500	0-16	0-1050	14-20	859-1202	0.75-0.87
C_{28}	5-17.5	500-1750	0-12	0-1350	10-14	997-1459	0.73-0.86
C_{30+32}	12.5-20	750-3500	25-40	0-2700	26-41	1411-2912	0.48-0.78
Comb. C_{24-32}	7.5-17.5	500-1750	7-16	0-1200	13-18	941-1396	C_{24}: 0.64-0.76
							C_{26}: 0.73-0.85
							C_{28}: 0.74-0.85
							C_{30+32}: 0.84-0.98
							Avg. Long chain: 0.75-0.88
